# A Novel Workflow for Non-Animal PBK Modelling of UV Filters: Oxybenzone as a Case Study

**DOI:** 10.3390/ph18111607

**Published:** 2025-10-24

**Authors:** Nazanin Golbamaki, Anne Moustié, Nicola J. Hewitt, Guillaume Lereaux, Matthew Burbank, El Mehdi Ben Yahya, Sébastien Grégoire, Laurène Roussel-Berlier

**Affiliations:** 1 L’Oréal, Research & Innovation, 93600 Aulnay sous-Bois, France; anne.moustie@loreal.com (A.M.); guillaume.lereaux@loreal.com (G.L.); matthew.burbank@loreal.com (M.B.); elmehdi.benyahya@loreal.com (E.M.B.Y.); sg@sebastiengregoire-consulting.fr (S.G.); laurene.roussel@loreal.com (L.R.-B.); 2 Cosmetics Europe, Avenue Herrmann-Debroux 40, 1160 Auderghem, Belgium; nickyhewittltd@yahoo.co.uk

**Keywords:** oxybenzone, dermal absorption, physiologically based kinetics models, PBK, cosmetic formulations, IVIVE

## Abstract

**Background/Objectives:** Physiologically based kinetics (PBK) modelling provides (internal) exposure concentrations. We used a PBK model parameterized exclusively with in silico and in vitro data in a bottom-up approach to predict the pharmacokinetics of oxybenzone, a UV filter, present in two formulations (for which dose-normalized C_max_ and AUC from clinical studies were different). **Methods**: Skin absorption data were used to refine chemical-specific dermal absorption parameters for oxybenzone in a lotion and spray. The Transdermal Compartmental Absorption and Transit (TCAT) model in GastroPlus^®^ 9.9 was used to estimate vehicle and skin layer diffusion and partitioning and then used to simulate systemic exposure. The model was validated according to the OECD 331 guideline. **Results**: PK profiles simulated for both formulations after single and repeated applications correlated with clinical data profiles (used only to validate our approach), with a deviation from the C_max_ and AUC of <2-fold. Sensitivity and uncertainty analyses indicated that most input parameters had a medium to high reliability, whereas only a few parameters related to dermal delivery had a low reliability: the partition coefficient between vehicle and water for spray and the diffusion coefficient in stratum corneum for lotion. In vitro skin absorption results suggested that absorption kinetics were not statistically different between the formulations; however, parameters such as vehicle evaporation time were different. The fine-tuned TCAT model containing the absorption data suggested that the variability in clinical data might be due to other factors, e.g., the small number of subjects. **Conclusions**: These results demonstrate how formulation-dependent absorption kinetics improve confidence in estimated exposure, thanks to the PBK model with its bottom-up approach for nonanimal-based safety assessments.

## 1. Introduction

Replacing animals in toxicological safety assessment requires a fundamental change from the current animal adverse-outcome-driven approach to a hypothetical, mode-of-action-driven framework. New approaches in toxicology based on in vitro methods and computational modelling offer considerable potential to improve the efficiency and effectiveness of chemical hazard and risk assessment in a variety of regulatory contexts. Physiologically based kinetic (PBK) modelling, a New Approach Methodologies (NAM)-based tool, computes and predicts the concentrations of a chemical (and its metabolites) within the body over time from a given external exposure. “Next generation risk assessment”, or NGRA [1], offers a way forward for animal-free safety decision-making, with principles specifically outlined for cosmetic ingredients, including detailed pharmacokinetics (PK) assessments. The use of NAMs and PBK for the safety evaluation of cosmetic ingredients is moving from a concept to practical use in NGRAs, with a focus on systemic toxicity [2]. PBK modelling is helping to bridge the gap between in vivo and in vitro evidence by providing data on (internal) exposure which can be compared with concentrations that were found to be active in in vitro assays [3,4].

Under the Cosmetics Europe Long Range Science Strategy (LRSS, 2016–2022), a series of seven read-across and eight ab initio NGRA case studies were performed to further the application of NAMs in addressing specific hypotheses for systemic toxicity assessment, inspired by the Seurat-1 framework and NGRA principles [5]. Read-across case studies focused on building confidence in the use of different NAM approaches for the selection of appropriate analogues with chemistry, toxicokinetics and the mode/mechanism of action/bioactivity insights. Ab initio case studies focus on assessing a chemical without reference to similar analogues and rely on applying available in vitro and in silico NAMs to identify levels of internal exposure and bioactivity to investigate the potential risk. A non-exhaustive list of methods used in the case studies were developed to characterize adversity/biological effects (e.g., cheminformatics, toxicogenomics, in vitro pharmacology profiling, organ-based functional assays, cell stress assays and in silico predictions) and characterize ADME properties (PBK modelling, in vitro ADME assays). The insights from Cosmetics Europe LRSS case studies aimed to advance the application of NAMs for the assessment of systemic toxicity potential of cosmetic ingredients. As part of this project, oxybenzone was one of the chemicals selected to run an ab initio case study, in which PBK modelling describing internal exposure was crucial in the safety decision. The generated data can also guide the design and rationale of in vitro tests performed for risk assessment and derive a so-called “Bioactivity Exposure Ratio” (BER), equivalent to a margin of safety (MoS), for decision-making [6,7].

PBK models describing the absorption, distribution, metabolism and excretion (ADME) of chemicals have a long history, stemming back to the 1930s [8]. Since the initial basic PBK models have been established, they are now used across different industries (e.g., pharmaceuticals, chemicals, pesticides, food additives, and cosmetics industries), as well as regulatory agencies (e.g., ECHA, EFSA, EMA), with increased complexity and application. PBK models must be built and validated using available PK data. The validated models can then be used for different purposes, including intra- and interspecies extrapolation, dose extrapolation, in vitro-in vivo and route-to-route extrapolations [9,10]. In the absence of PK data, “bottom-up models” can be built based on in vitro- and/or in silico-derived ADME properties. The validation of such models is a real challenge and remains an active research area [11].

In the cosmetics industry, the use of PBK modelling can impact the regulatory approval process for cosmetic ingredients in several ways, e.g., by helping regulatory agencies to make more informed decisions by providing robust quantitative data, predicting internal systemic concentrations of ingredients or their metabolites, and by identifying the target tissues or organs most likely to be targeted for a more accurate safety assessment.

PBK models are used by the pharmaceutical industry in various stages of drug development [12], and these are calibrated and validated using in vivo clinical PK data. In this regard, the use of PBK modelling in cosmetics regulatory decision-making poses significant challenges. Indeed, the use of such approaches within the cosmetics risk assessment arena is still dependent on whether the model is validated against in vivo data. On one hand, animal testing has been banned for cosmetics ingredients and products since 2013 [13], thus no in vivo PK data can be generated for these chemicals. On the other hand, the Scientific Committee on Consumer Safety (SCCS), which provides opinions on the health and safety risk assessment of cosmetics ingredients in the EU, emphasizes that, at present, no validated alternative methods (non-animal) that completely cover the field of ADME exist [14,15]. Indeed, the SCCS highlights that the acceptable prediction of dose metrics should follow the acceptance criteria outlined in the World Health Organization (WHO) guidance [16]. Therefore, it should be demonstrated that the model correctly predicts experimental data that has not been used to build the model (i.e., the ratio between simulated and observed data should be on average within a factor of 2). This emphasizes the need to develop effective ways to validate these models and define their uncertainty. Indeed, the feasibility of developing and validating PBK models based only on in silico and in vitro data (in vitro to in vivo extrapolation; IVIVE) is a topic of major interest for both regulators and the scientific community. The Organization for Economic Co-operation and Development (OECD) 2021 Test Guidance 331 [17] emphasizes in vitro–in silico use for PBK model development without in vivo PK data or limited in vivo data. In this paper, we propose a workflow to develop a dermal PBK model for UV filter applied topically, parameterized entirely using data obtained from in vitro and/or in silico methods in a bottom-up modelling approach and using Franz cell skin absorption data to refine dermal parameters with a focus on formulation effects.

Two human dermal PBK models for two distinct commercial formulations were developed using the example of oxybenzone, a well-known UV filter. The model was built according to the OECD Test Guidance [17]. The external model validation was performed by comparing simulated values with clinical data (not used for parametrization of the model), which were available for this ingredient from studies reporting the plasma concentration profiles of oxybenzone measured after the application of sunscreens under maximal use conditions [18,19].

GastroPlus^®^ 9.9 software TCAT model was developed to predict drug disposition in vivo after the topical or subcutaneous application of drugs in different formulations [20,21]. TCAT is based on a mechanistic dermal model which considers diffusion and partition across vehicles, different layers of skin (i.e., stratum corneum, viable epidermis, and dermis), and systemic circulation. Diffusion and partition coefficients can be measured experimentally [22] or estimated by the model. TCAT includes several literature-based options for calculating these parameters: Wang–Kasting–Nitsche [23,24], Potts–Guy [25], and Robinson [26] for the stratum corneum; Bunge–Cleek [27] and Robinson [26] for viable epidermis; and Ibrahim [28] for a first-order model for the systemic absorption rate from the dermis.

Knowledge of the dermal absorption of a UV filter is critical for safety assessment but is complicated by the variability in formulation effects [29]. Nonetheless, the effect of various formulations on the skin absorption of UV filters was studied in previous similar works with a bottom-up approach [30]. Hamadeh et al. [31] recently developed a dermal model for oxybenzone using in vitro data from a radiolabelled skin penetration assay. However, these data were acquired using application amounts [32] and formulations which were different from those in the clinical studies used for model validation, thus potentially compromising model development. Moreover, unlike our current approach, the authors employed clinical data for both model refinement and validation—not as an alternative to the use of the in vivo data approach. Therefore, we used a framework employed by others [30], whereby several levels (L) of increasing complexity and refinement were explored, depending on how the models were parameterized: (L1) in silico only, (L2) in silico and in vitro, and (L3) in silico, in vitro and clinical data.

Dermal model parameters were categorized as either formulation-specific or chemical-specific. The formulation (i.e., vehicle) affects the kinetics of a key phase of the TCAT model, i.e., the release of the chemical from the vehicle into the stratum corneum. Consequently, the vehicle evaporation rate and chemical diffusivity within the vehicle affect both the diffusivity within the stratum corneum and the partitioning between the vehicle and the stratum corneum. Conversely, chemical-specific parameters govern chemical diffusivity and partitioning through the viable epidermis and dermis. The innovative aspect of the current study is that we have harmonized the chemical-specific parameters for two formulations. To ensure consistency in our analysis, we took an average of the values obtained experimentally for both models. Another approach proposed in the workflow was to address the optimization process in cases where chemical-specific parameters are needed, whereby data across all formulations can be used to refine chemical-specific parameters. This approach is essential for fine-tuning absorption predictions, whereby leveraging more kinetic data points from different formulations will facilitate better optimization.

Here, we describe the different stages of the model development that aimed to validate the model according to the OECD’s guidance [17]. Moreover, the effect of the two formulations of oxybenzone based on in vitro and clinical data were analyzed. We then simulated the plasma concentrations by focusing on the highly sensitive and uncertain parameters to validate the formulation effect versus population variability in the UV filter absorption measured in clinical studies.

## 2. Results

**Figure 1:**
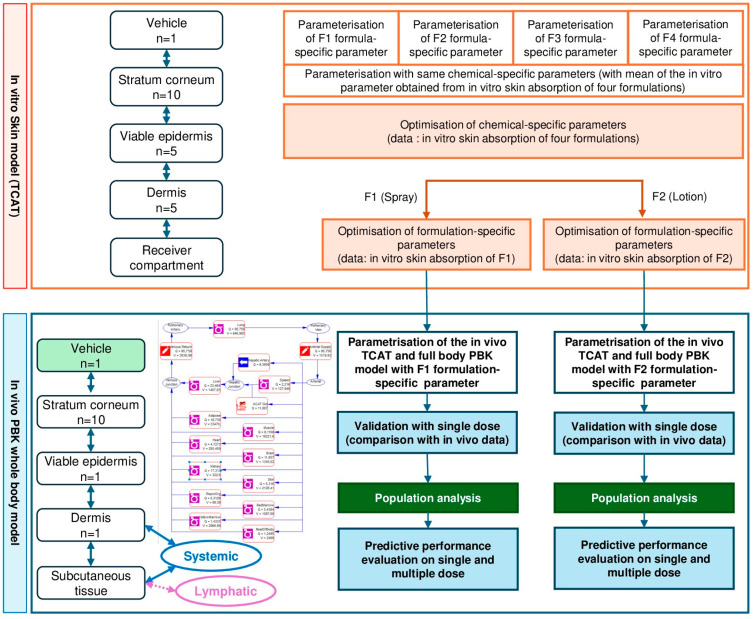
Steps for PBK model development and TCAT refinement. Note that optimization, validation and performance were performed on single dose administration. Predictions are performed for 500 subjects to take the population variability into account. The uncertainty of values obtained in vitro and in silico are also integrated in this analysis (See Supplementary Materials Section S1.5).

An overview of the workflow used to build the oxybenzone PBK model is shown in Figure 1.

### 2.1. In Vitro Skin Absorption and Evaluation of the TCAT Model for In Vitro Skin Absorption

The conditions of the skin penetration test were fully in line with the conditions specified in the in vivo clinical studies used for PBK model development. This included the application of 2 mg/cm^2^ on the upper skin surface (stratum corneum). This condition was not respected in a PBK model developed by other authors [31], where much higher infinite doses were used, e.g., 56.5 mg/cm^2^, as the input data for the dermal model. The absorption of oxybenzone was not significantly different for dose-normalized formulations according to N-Way ANOVA unpaired value analysis [32]. Indeed, quantities identified in the stratum corneum, viable epidermis and dermis, as well as the receptor fluid, for all formulations were not significantly different (Supplementary Figure S1B).

**Figure 2:**
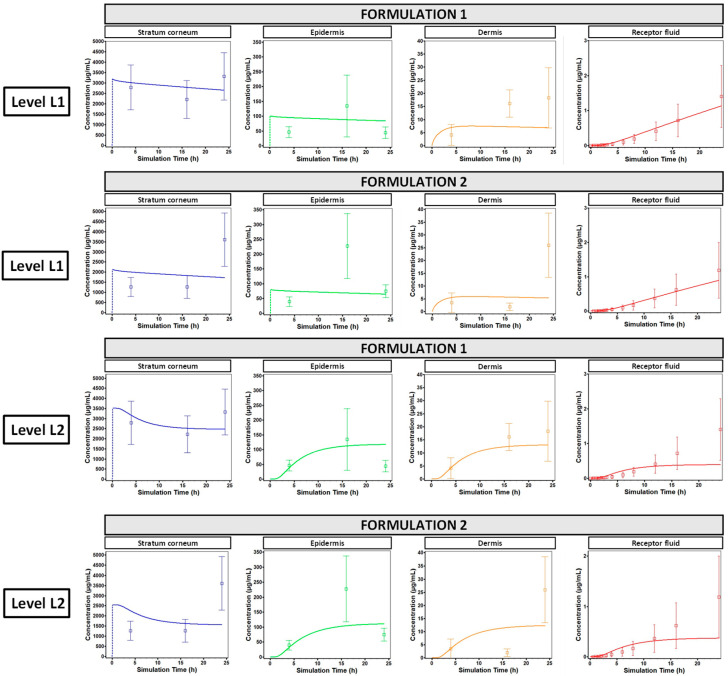
TCAT model output after optimization based on highly sensitive parameters. Panels represent the simulation of in vitro penetration of oxybenzone across different layers of human skin (blue: stratum corneum, green: epidermis; orange: dermis; and red: receptor fluid) for Formulations 1 and 2. Input parameter values for the simulation are listed in Supplementary Table S1. Solid lines represent predicted values, square symbols with associated error bars represent measured values in in vitro skin absorption experiments (mean ± SD, n = 8).

Figure 2 shows the results of the refined in vitro TCAT model with respect to the predicted and measured concentrations of oxybenzone in different layers of the skin and the receptor fluid after application of the two formulations. This was performed by parameterizing TCAT with in silico parameters only (L1) and then comparing the outcome with that after parameterization of the model with in vitro data (L2). In both parametrization approaches, the highly sensitive parameters were optimized against skin absorption data.

### 2.2. Prediction of Human Plasma Concentrations

**Figure 3:**
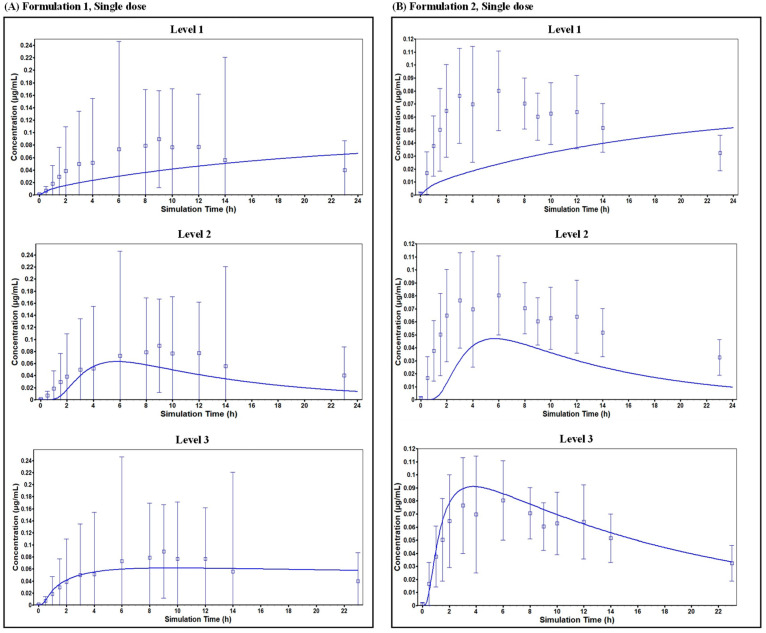
Comparison of measured and simulated in vivo plasma concentrations after a single application of oxybenzone present in (**A**) Formulation 1 and (**B**) Formulation 2. Mean predicted values for different levels of the PBK framework are shown as a continuous blue line and measured values are denoted by square symbols and error bars.

**Figure 4:**
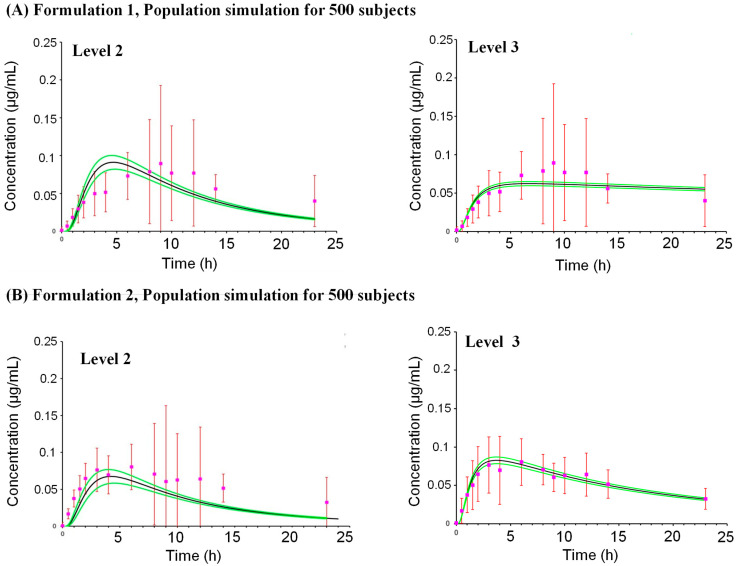
Comparison of measured and simulated in vivo plasma concentrations after a single application of oxybenzone present in (**A**) Formulation 1 and (**B**) Formulation 2 in a population of 500 human subjects. Measured values are denoted by square pink symbols and error bars. Mean predicted values for levels 1 and 2 of the PBK framework are shown as a continuous black line and the green lines denote the lower and upper 90% interval confidence.

**Figure 5:**
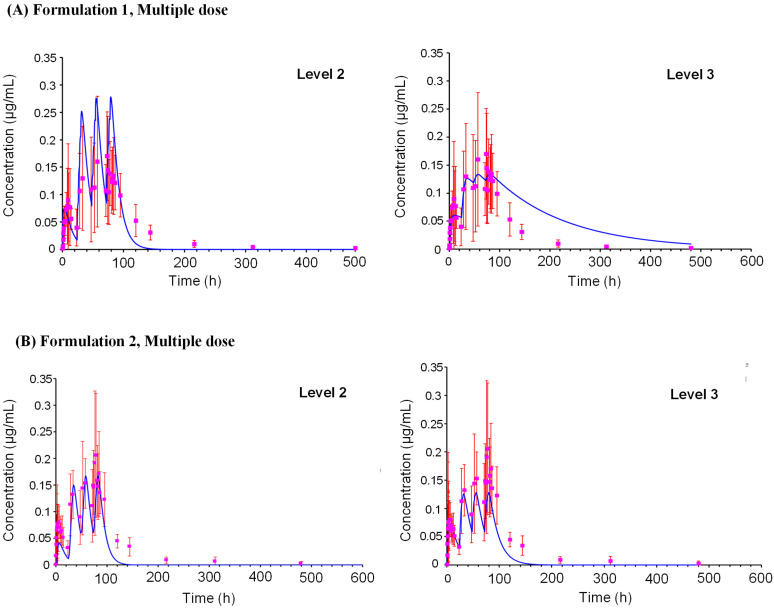
Simulation of in vivo plasma concentrations of oxybenzone over time after repeated topical application. Plasma concentrations after repeated topical application of (**A**) Formulation 1 and (**B**) Formulation 2 are according to the clinical trial scenarios. Simulations were according to the demographics of individual subjects (age, BMI, sex) in the clinical trials. Predicted values are shown as a continuous blue line and measured values are denoted by pink square symbols and error bars.

The PK profiles of oxybenzone were predicted for Formulation 1 and 2, which were then compared with observed clinical data (Table 1). For Formulation 1, after a single application, the predicted time to maximum concentration (T_max_) was ~5.75 h using the L2 prediction, which was earlier than the observed T_max_ of 9 h (Figure 3). However, the predicted plasma concentration profile between 6 and 24 h was comparable to the observed profile. The predicted maximum plasma concentration (C_max_) and area under the curve (AUC) ratios (predicted/observed) were 0.71 and 0.63 using the L2 predictions, respectively. A simulation for a population of 500 subjects (Figure 4) predicted a mean C_max_ of 0.0982 µg/mL (CV% 150.99) with a 90% confidence interval ranging from 0.087 to 0.109 µg/mL. After multiple applications (one application on Day 1, followed by four applications per day on Days 2–4 with a 2 h interval) (Figure 5), the L2 predicted C_max_ was 0.278 µg/mL and AUC_0–t_ was 14.6 µg·h/mL, resulting in predicted/observed ratios of 1.64 and 0.94, respectively.

For Formulation 2, a single application resulted in a better prediction of the plasma concentration profile. The L2 predicted C_max_ ratio was 0.585 and the AUC_0–t_ ratio was 0.473 when compared to observed values. A simulation for a population of 500 subjects predicted a mean C_max_ of 0.07788 µg/mL (CV% 153.79) with a 90% confidence interval ranging from 0.069 to 0.087 µg/mL. Following multiple applications (the same regimen as Formulation 1), the L2 predicted C_max_ was 0.270 µg/mL and AUC_0–t_ was 13.5 µg·h/mL, yielding predicted/observed ratios of 1.29 and 0.80, respectively.

Overall, the L2 models effectively predicted the PK profile of oxybenzone for both formulations, with C_max_ and AUC ratios generally between 0.4 and 1 across different dosing schedules and prediction levels. While a single application of Formulation 1 resulted in an underestimation of T_max_, the predicted and observed plasma concentration profiles aligned well between 6 and 24 h.

### 2.3. Model Performance

A sensitivity and uncertainty analysis was performed on the parameters used to simulate C_max_ after a single application of Formulations 1 and 2.

#### 2.3.1. Formulation 1

Sensitivity: The most sensitive parameters were the vehicle partition coefficient (K_VH/w_) and ratio of blood to plasma concentration (Rbp). The dermis diffusivity (D_DE_), dermis/water partition coefficient (K_DE/w_) and the stratum corneum diffusivity (D_STCOR_) showed medium sensitivity. All other parameters showed low sensitivity.

Uncertainty: All parameters demonstrated medium to low uncertainty. The only parameter with high uncertainty was K_VH/w_.

Reliability (Table 2(A)): K_VH/w_ showed low reliability. All other parameters had medium to high reliability.

#### 2.3.2. Formulation 2

Sensitivity: D_STCOR_, K_VH/w_, D_DE_, K_STCOR/w_ and K_DE/w_ as well as Rbp were highly sensitive parameters. All others were low sensitive parameters.

Uncertainty: D_STCOR_ had the highest uncertainty. All other parameters displayed medium uncertainty.

Reliability (Table 2(B)): D_STCOR_ had low reliability. The remaining parameters demonstrated medium to high reliability.

In summary, dermal delivery parameters, particularly parameters related to the vehicle and stratum corneum (formulation-specific), were crucial for both formulations. The K_VH/w_ presented the greatest uncertainty and lower reliability for Formulation 1. The D_STCOR_ presented the greatest uncertainty and lower reliability for Formulation 2.

The output (C_max_) of the two models was sensitive mainly to Rbp and K_VH/w_. Only Rbp and K_VH/w_ were highly sensitive for Formulation 1, whereas for Formulation 2, other parameters such as D_STCOR_, K_STCOR_, D_DE_, and K_DE/w_ were highly sensitive parameters, potentially impacting its predicted C_max_.

## 3. Discussion

Given the restrictions on animal testing, an accurate prediction of human exposure is critical. To address this directly, and without recourse to in vivo animal data, we developed novel PBK models incorporating only in vitro or in silico data to evaluate the systemic exposure to two oxybenzone-containing sunscreens. These were tested in a clinical trial reported by Matta et al. [18], in which oxybenzone was applied as a lotion or a spray. Interestingly, our analysis revealed significantly greater systemic absorption of oxybenzone from the lotion despite the spray containing 50% less oxybenzone, underscoring the potential of formulation effects to be investigated as opposed to the possible variability in the clinical trial resulting from the small number of participants.

The developed PBK models with a combination of in silico and in vitro bottom-up parameterization (i.e., L2) exhibited strong predictive performance, falling within 2-fold of the clinically observed AUC and C_max_ values. This necessitated the refinement and validation of the PBK model, particularly exploring the mechanistic basis of the formulation-dependent differences by investigating formulation-dependent parameters such as vehicle and stratum corneum diffusivity and partition coefficients.

### 3.1. Development of the Skin Module

The TCAT model was refined using data derived from in vitro skin absorption assays for two distinct formulations containing oxybenzone. As noted by Grégoire et al. [33], the type and quantity of input data used in the different in silico skin absorption models can vary considerably. Indeed, many parameters can influence the kinetics of dermal absorption, including the formulation [34,35,36,37]. The equations and assumptions behind the software can impact its predictivity, which in turn, can introduce uncertainty. For example, for the IVIVE, the underlying biochemical, mathematical, and physiological bases are all important in defining the performance of the model [38]. The dermal delivery using frozen skin was relatively well predicted for a finite dose of aqueous solution by the complex in silico models evaluated by Grégoire et al. [33] (including the TCAT model). Having refined the TCAT model using in vitro parameters linked to formulation-specific parameters, there was a good correlation between simulated and measured in vitro skin distribution and penetration kinetics of oxybenzone applied in both formulations. The current TCAT model version lacks an output plausibility check for optimized parameter values. To address this lack of predefined boundaries, the optimization settings incorporated the highest and lowest experimentally reported values, thus avoiding potentially unrealistic parameter values [22].

A review by Scheuplein and Blank [39] provides a detailed discussion of the resistance to diffusion in the various skin layers. The authors explain that the stratum corneum is the primary barrier to diffusion, with the viable epidermis and dermis offering much less resistance. This information can be used to inform the interpretation of the simulation results and the optimization of the model parameters. Although the simulated concentration profiles in different skin layers were meeting the experimental values resulting from the in vitro absorption test, the partition coefficient and diffusivity in the skin layers did not support the hypothesis that the stratum corneum is the layer posing the highest resistance. Indeed, the model produced similar trends when assessing the available literature for three UV filter ingredients. In other words, the predictions (simulated concentrations in each skin layer) match the experimental output, but the generated partition coefficient and diffusivity values support the historical approach for assessing the resistance of the skin layers. It should be noted that the model considers such values to be constant albeit inherently variable. In addition, the exposure time is currently set to evaporation time while this might not be the case. However, setting the exposure to 24 h did not result in simulations that could match the experimental value points resulting from the in vitro absorption tests.

### 3.2. Evaluation of the Sensitivity Analysis

A sensitivity analysis of PBK models can highlight the most influential parameters, which are generally skin-related parameters for models simulating the dermal route [40]. This study identified K_VH/w_ and Rbp as the most sensitive parameters for predicting C_max_ for both formulations. For Formulation 2, D_STCOR_, K_STCOR/w_, D_DE_, and K_DE/w_ were also highly sensitive. For Formulation 1, the C_max_ is slightly underestimated using the L3 prediction—this model is optimized using clinical data, which are much more variable for Formulation 1. For Formulation 2, the L1 and L2 predictions underestimated C_max_, while the L3 prediction model slightly overpredicted C_max_. The sensitivity analysis parameters related to the whole body (excluding the skin) identified Rbp as the most sensitive parameter for both Formulations 1 and 2. However, the relative sensitivity of parameters related to skin absorption (after Rbp) for the two formulations are different: For Formulation 1, the order of sensitivity is the vehicle layer partition coefficient followed by the dermis diffusion and dermis partition coefficient; for Formulation 2, the stratum corneum diffusion is the most sensitive, followed by the dermis diffusion and dermis partition coefficient. This highlights how these two Formulation models can be affected differently, i.e., by the vehicle layers value in Formulation 1 and by the stratum corneum diffusion in Formulation 2.

Physiological parameters did not significantly influence predictions, and the blood/plasma concentration ratio (Rbp) was the only parameter that impacted distribution, exhibiting high sensitivity for both formulations. The solvent residual volume fraction sensitivity resulted in only moderate reliability for Formulation 2, which suggests the potential for refinement in future simulations. Overall, for both formulations, the model showed medium to high reliability with respect to all parameters except for K_VH/w_ in Formulation 1 and D_STCOR_ in Formulation 2, showing the importance of improvement in the confidence in these parameters.

### 3.3. Uncertainty Evaluation

Uncertainty is a subjective assessment of how reliable the input parameters are. A formal uncertainty analysis as suggested in the WHO PBPK guidance is difficult to perform with a bottom-up PBK model, as the ratio of median to 95th percentile reflects a measure of variability rather than true uncertainty obtained when the PBK model parameters are fitted to an observed dataset. The uncertainties of predicted parameters were higher than derived from experimental values. Moreover, as mentioned in the SCCS guidance of 2021 [14], the use of estimated values in further modelling might increase uncertainties associated with a model. Based on this assumption, the uncertainty of parameters that were optimized based on ex vivo skin absorption data were increased (from low to medium).

It should be kept in mind that besides the uncertainty in the input data (in vitro, in silico) used for the model, other sources of uncertainty exist. Indeed, the OECD Test Guidance addresses uncertainties in the assessment of PBK modelling for regulatory application and separates it into three categories: (1) uncertainties in the model input parameters (i.e., variability in the parameter estimates due to intrinsic biological variation or measurement error); (2) uncertainty in the model algorithm/structure (biological basis) and (3) uncertainty in the model output(s), derived from uncertainties in the input parameters and model structure. The guideline suggests that some uncertainties are not possible to reduce, e.g., inherent variability in a value due to inter-individual variation, whereas other uncertainties, such as the equations forming the basis of the model or measurement errors, can be reduced. Regarding the reliability of the parameters used in our study, some parameters could be improved, e.g., Rbp, K_VH/w_, K_DE/w_, or D_DE_. Parameters related to the skin are critical and therefore need the development of adapted protocols to refine their measurement. Another parameter related to the skin, ksys (systemic absorption rate), has been reported to be paramount [28].

### 3.4. Formulation Effects

An important feature of any dermal absorption in silico model used for safety assessment purposes is the ability to reflect formulation effects. When developing models for different formulations, there are several in silico and in vitro data which are formulation-specific [34,37,41]. After topical exposure of a finite amount of formulation, the composition of the formulation on the skin varies as a function of time with ingredient penetration and evaporation. This means that the chemical concentration at the skin surface increases over time (until it reaches the solubility limit in the remaining vehicle), which affects the diffusion coefficient in the stratum corneum and the partition coefficient between stratum corneum and vehicle. The TCAT model assumes constant partition and diffusion coefficients, which may result in an over- or underestimation of the skin absorption. To overcome this constraint, water was selected as the vehicle in the TCAT model. Generating in vitro values that accurately reflect the inherent inconstance of these values remains an area requiring further understanding and investigation. The software (version 9.9) does not handle the dry down of the formulation on the skin. The skin hydration state depends on the model’s settings when using the available in silico options. When using the Wang–Kasting–Nitsche model [23,24], the software provides a choice of using full or partial skin hydration. For this work, experimental values for stratum corneum diffusivity and partitioning were used, and it was assumed that there were partial hydration conditions since the experiment was not performed under occluded conditions.

Formulation effects on the absorption of a chemical are often a critical consideration in this perspective and comparative studies, such as ours, can shed light on these aspects. The PBK model incorporated the formulation characteristics, which assisted in understanding their potential impact on plasma concentrations. Predicted C_max_ values were approximately 0.1 µg/mL (with the L2 parameterization) for both Formulations 1 and 2, with AUC_0–∞_ values ~14–15 µg.h/mL in the population analysis. While the TCAT model predicted faster skin penetration of oxybenzone when applied in Formulation 2 compared to Formulation 1, the whole body PBK model simulations showed similar C_max_ and AUC values for both formulations. This aligns with in vitro experiments, where oxybenzone absorption profiles were comparable across formulations.

The differences in plasma concentrations observed in the clinical study after the application of Formulation 1 and 2 could be related to variability in systemic parameters, e.g., Rbp (which was identified as a sensitive parameter). These parameters do not play a role in the absorption of oxybenzone across the skin, which could explain why similar formulation effects were not observed in the in vitro assays. The application conditions in clinical settings are critical but are less controlled than those for in vitro studies. It was not possible to evaluate these in depth as the clinical study report does not specify the application conditions, other than the amount applied to the body surface. The subjects had to remain still for an entire day to avoid removing the sunscreen from the surface of the skin, which may not have been strictly adhered to, thus resulting in variability. In addition, friction caused by clothing can also contribute to the variability in the amount that remains on the body after application. In addition, other factors could have influenced the variability in absorption between the two formulations, such as the skin surface area to which the sunscreen was applied, which may differ between subjects (especially if there are marked differences in the BMI, as was the case in the clinical study).

### 3.5. Use of In Silico, In Vitro and Clinical Data

The predictive capacity of the full body PBK model parameterized by L1 with respect to the AUC_0-∞_ and systemic clearance (Cl_sys_) is low and not representative of the kinetic profile in the observed values. This emphasizes the need to incorporate in vitro data to result in a more reliable prediction of systemic exposure. For L2 parameterization, the predictive capacity was very good, with comparable PK profiles for the different formulations and only a small deviation from the measured values of less than 2-fold. According to the WHO guidelines, among other criteria, a model can be validated if the ratio is <2 [16] (discussion around the validity of this threshold is out of the scope of the current paper). This is significant in this study because the predictions were conducted using only in vitro data and the model was refined using chemical- and formulation-specific parameters. The very good performance of this model may be chemical-specific such that certain characteristics of oxybenzone favour a good prediction of its PK profile—highly lipophilic and not metabolized in the skin. It is well established that metabolism can take place in the skin [42] and this could modify the level of skin absorption particularly for lipophilic chemicals [43,44]. The metabolism of oxybenzone in the skin is negligible, thus reducing uncertainty due to first-pass metabolism as it penetrates the skin. Nevertheless, other sunscreen ingredients (e.g., homosalate) are hydrolyzed within the skin [40] (even in frozen in vitro skin), which impact the overall PK profiles [22,28,39].

A notable observation was that the population analysis resulted in a higher concordance between the predicted and observed mean C_max_ values for both formulations compared to that for a single individual. A population analysis considers the variability that is expected for each of the input parameters, including the vehicle layer partition coefficient and the stratum corneum diffusion. While traditionally PBK models are built for one individual, the population analysis captures the variability that can be observed in vivo. Therefore, the mean predicted values resulting from population analysis are more indicative of clinical mean values.

### 3.6. Use of PBK Models in NGRAs

In NGRAs, the challenge for the PBK modelling is to parameterize models based on data obtained from in vitro and/or in silico methods, with limited or no availability of in vivo kinetic data for dermal absorption routes to calibrate the models and validate the model outputs. These data should be of good quality and can further help the design and rationale of in vitro tests performed for risk assessment and derive a BER or MoS for regulatory purposes. Indeed, estimated data from PBK models are intended to be used for MoS calculation, i.e., for quantitative safety evaluation [15]. Currently, from an NGRA perspective, there is no universal consensus on which single parameter to use for risk assessment, as the most relevant choice often depends on the specific toxicological profile and mechanism of action of the ingredient. C_max_ represents the peak plasma concentration of a cosmetic ingredient after application, indicating the highest level of exposure at a specific time point. AUC represents the total exposure to the ingredient over time, reflecting the cumulative amount absorbed and the duration of exposure. Both parameters are crucial in evaluating the safety and efficacy of cosmetic products by assessing ingredient absorption and bioavailability.

Regarding oxybenzone, legacy in vivo data provided as part of the dossier were evaluated by the SCCS [45]. For the calculation of the MoS, the SCCS used an in vivo NOAEL of 67.9 mg/kg bw/day from the Nakamura et al. [46] study, in which effects of oxybenzone were observed on the development and reproductive organs of offspring of time-mated female Harlan Sprague-Dawley rats. The SCCS calculated an MoS of 38 for the use of oxybenzone at a concentration of 6% as a UV filter in sunscreens for whole-body application. For read-across and ab initio NGRAs for oxybenzone, efforts are underway to characterize the bioactivity of oxybenzone using in vitro, in chemico or in silico models (initiated during the Cosmetics Europe Long Range Science Strategy programme [47] and continued under the ongoing ICCS (International Collaboration on Cosmetic Safety).

## 4. Materials and Methods

### 4.1. Test Chemical Properties and Available Clinical Data

The UV filter, oxybenzone, was used to demonstrate our approach in the dermal PBK model development with a focus on formulations in various products. Physicochemical properties of oxybenzone (CAS number: 131-57-7) sourced from an ADMET predictor 10.3.0, were as follows: molecular weight: 228.2 g/mol, strongest acid pKa: 10.11, solubility in water (Sw): 0.071 mg/mL, or measured octanol–water partition coefficient (LogP): 3.38 (median of two experimental values [48]).

Clinical data were available for this ingredient from two studies reporting the plasma concentrations profiles of oxybenzone measured after dermal application of the sunscreens under maximal use conditions [18,19], herein denoted as Dataset A and B:

Dataset A: Formulations 1 and 2 were applied once on the first day and plasma concentrations of oxybenzone were measured for each participant over a one-day period. The study was continued to four applications per day every 2 h for three days [18].

Dataset B: Formulations 2, 3 and 4 were applied four times daily at 2 h intervals for four days and plasma concentrations of oxybenzone were measured for each participant over a seven-day period [19].

In these clinical studies, oxybenzone was present in different formulations in the following commercial products (details of these formulations are reported by Hamadeh et al. [31]):

Formulation 1: 6% oxybenzone in Aerosol Spray (Aerosol Spray in Matta et al. [18].

Formulation 2: 4% oxybenzone in Lotion (lotion in Matta et al. [18,19]).

Formulation 3: 6% oxybenzone in Spray (Spray 1 in Matta et al. [19])

Formulation 4: 5% oxybenzone in Spray, (Spray 2 in Matta et al. [19]).

Clinical studies were compared to see if there was a difference in dose-normalized absorptions, i.e., C_max_ divided by the oxybenzone dose (according to the % in the formulation). The results showed that despite the lower concentration (up to 50%) in the lotion (Formulation 2), there was a statistically significantly higher systemic absorption compared to the spray (Formulation 1) (Supplementary Figure S1A). For the development of the dermal PBK model, we focused on these two formulations, i.e., the most distinct formulations. Notably, all four formulations were used to optimize the chemical-dependent parameters in the skin. Additionally, the two formulations were reported in the same clinical trial with the same application scenario, including a single application for 24 h followed by repeated application over three days, published in 2020, reducing the variability. The observed variability was then investigated with robust studies on larger sample sizes using PBK modelling. All clinical values were used for the validation of the PBK model and none of the outliers in the clinical study were excluded, even if they resulted in a high inter-individual variability (e.g., CV > 150%).

### 4.2. Dermal Disposition and Absorption Data

#### 4.2.1. In Vitro (Ex Vivo) Skin Absorption Data

This study used abdominal human skin samples from anonymous female donors obtained from plastic surgery procedures. French regulations (article L. 1243-4 of the French Public Health Code) and Declaration of Helsinki Act did not require the study to be reviewed or approved by an ethics committee because patients’ written informed consents were collected and kept by the surgeon. Only the age, sex and anatomical site of samples were specified to the authors. The authors did not participate in sample collection.

The skin absorption of oxybenzone was measured according to the OECD Test Guideline 428 describing skin absorption using in vitro human skin [49]. Fresh human skin was ethically obtained with donor consent from female donors (n = 8) undergoing abdominal surgery. The skin was stored at −20 °C before use. The frozen skin was thawed and then cut to a mean thickness of 907 ± 246 µm. This is out of the 200–400 µm range recommended in the OECD Test guideline 428 [50]; however, the Gastroplus 9.9 software, TCAT module, for which this data was generated, is designed to include an input value for skin thickness and thus accounts for the exact skin thickness measured in the experiment. Cutting very thin skin layers led to folding and damage of the skin; therefore, thicker slices were used, which were still within the acceptable thickness range for full-thickness skin (<1 mm). The skin was maintained at a temperature of 32 ± 1 °C in a static Franz diffusion cell (Nebo, Les Lilas, France). The acceptance criterion for an experiment was based on transepidermal water loss (TEWL), measured after 1 h of equilibration (measured using a Delfin VapoMeter^®^ (Delfin Technologies Ltd., Kuopio, Finland)), which was always <20 g/h/m^2^.

A quantity of 4 mg of each formulation was applied on the skin surface (i.e., 2 cm^2^), yielding a specific dose of 2 mg/cm^2^, representative of the quantities applied in the clinical data. This quantity is in accordance with regulations set out by the SCCS [15]. The receiver chamber below the skin contained 3 mL of receptor fluid (RF), which was stirred with a magnet bar. The RF (phosphate-buffered saline + 4% Brij O20, INCI: polyoxyethylene (20) oleyl ether) was collected at various timepoints after application over 24 h (0.5, 1, 1.5, 2, 2.5, 3, 4, 5, 6, 8, 10, 12, 16, 20, and 24 h). The RF was partially (300 µL) replaced with fresh buffer after each time point. After 4, 16 and 24 h, the surface of the skin was washed with one cotton-tip soaked with isopropanol, followed by skin drying with two cotton-tips for the four formulations. The stratum corneum was removed by tape stripping with a maximum of 20 strips performed for each skin sample. The strips were pooled (into 5 strips per pool) and extracted in a suitable solvent (methanol or dimethyl sulfoxide/methanol 50/50 *v*/*v*). The epidermis and dermis in the application area were cut out using a scalpel blade and then separated by heating. To separate the layers, samples were placed in a plastic bag and then immersed in a water bath at 60 °C for 2.5 min. The epidermis and dermis were then separated using a scalpel and placed in a flask and weighed. Oxybenzone was extracted from the epidermis and dermis using a suitable solvent (dimethyl sulfoxide/methanol 50/50 *v*/*v*). All samples were analyzed for oxybenzone by LCMS/MS (see analytical methods in Supplementary Materials Section S1.1).

In vitro skin absorption data (see Supplementary Figure S1B) were used to derive partition coefficient values based on the ratio of oxybenzone in the skin layers, i.e., K_STCOR/VH_ K_VE/STCOR_ K_VE/DE_ and K_RF/DE_.

#### 4.2.2. Kinetics in Isolated Dermis

The skin samples used to measure kinetics in isolated dermis were from the same donors as those used for measuring in vitro skin absorption (n = 8). The epidermis and dermis were separated by heating (in a bag immersed in a water bath at 60 °C for 2.5 min). The dermis was maintained at 32 ± 1 °C on a static Franz diffusion cell (Nebo, Les Lilas, France). A volume of 2 mL of a saturated aqueous solution was applied to the surface of the dermis (2 cm^2^). Albumin was added to the vehicle but not to the RF to be closest to the physiological conditions (as recommended by Hummer et al.) [51]. The RF was collected at various timepoints after the application over 24 h (1.5, 3, 5, 7, 9, 12, 14, 16, 20, and 24 h) and the volume removed was replaced with fresh buffer after each time point. All samples were analyzed by LCMS/MS (according to the method in Supplementary Materials Section S1.1). The kinetics on isolated dermis data were used to calculate the diffusion coefficient in the dermis: D_DE_. For this, the second law of Fick was used [52]:

(1) $$Q_{rf}=m1\left[m2*t-\frac{1}{6}-\frac{2}{\pi^{2}}\sum_{n=1}^{\infty }\left(\frac{{\left(-1\right)}^{n}}{n^{2}}\;*\;exp\left(-m2\;*\;n^{2}\;*\;\pi^{2}\;*\;t\right)\right)\right]$$

With

(2) $$m1=C_{d}\;*\;\left(K\frac{D_{DE}}{Veh}\;*\;L\right)$$

(3) $$m2=\frac{D}{L^{2}}$$

where K is the partition coefficient between dermis and vehicle, D_DE_ is the diffusion coefficient in dermis, L is the dermis thickness, C_d_ is the applied concentration, t is the time, and n is the integer. The cumulated amount per unit area of dermis (Q_rf_) was plotted as a function of time (t) using GraphPad (Prism 7.05) software to determine the parameters m1 and m2.

#### 4.2.3. Thickness and Volume of Skin Layers

The thicknesses of the epidermis and dermis used for the TCAT in vitro module were measured indirectly. The skin was weighed (during the in vitro skin absorption experiment) and the weight was converted into volume assuming a density of 1 and then converted in thickness using the actual surface area of 2 cm^2^. The thickness of the stratum corneum from each donor sample was measured by Optical Coherence Tomography (OCT Light-CT scanner, LLTech, Paris, France) [53] (See Supplementary Table S5 for the values for each donor). For the TCAT in vivo module, the layers’ thickness (stratum corneum, epidermis, and dermis) were set to Gastroplus 9.9 default values.

#### 4.2.4. Residual Volume Fraction

Evaporation kinetics of the formulation are required as an input for the TCAT model. To measure this parameter, 4 mg of the UV filter formulation was applied to a delimited surface of 2 cm^2^ on a blade. The laboratory temperature was maintained at 22 °C ± 2 °C and the humidity was 50 ± 10% (these values were checked during the experiment). The exact applied amount was determined by weight. The blade was left uncovered at room temperature on the balance during the entire experiment, during which the weight was recorded according to the elapsed time (5 min, 10 min, etc.) until it was stable (a few hours). This defined the percentage of vehicle residual volume. The GastroPlus 9.9 software calculates the corresponding evaporation rate, from which it calculates the evaporation time using Equation (4) by taking into account the Residual Fraction (obtained in laboratory) and the dose volume [54]:

(4) $$EvapTime=\frac{Dose\;volume\;*\;(1-Residual\;Fraction)}{Evaporation\;Rate}$$

The Peress equation does not need a value for the solvent air diffusivity to calculate the evaporation rate and evaporation time. Therefore, it was used to simplify the calculations.

The calculated evaporation time was then reported as the application time to consider the volatility of the formulation. It was assumed that after evaporation, oxybenzone was not able to penetrate the skin.

#### 4.2.5. Skin Metabolism Data

Skin metabolism was assessed using skin S9 prepared from reconstructed human epidermis Episkin^®^ (EPISKIN SA a subsidiary of L’oréal, Lyon, France), as described previously [55]. The studies by Genies et al., [42] confirmed that these skin models exhibit functional metabolizing enzyme activities similar to those in native human skin, making them useful tools for evaluating metabolism of test chemicals. The incubations were conducted using 5 µM oxybenzone for up to 240 min. Control samples without S9 showed that oxybenzone was stable at 98% of the initial concentration over the entire incubation time. The half-life of oxybenzone was 856 ± 215 min, corresponding to a skin clearance of 0.40 ± 0.14 µL·min^−1^·mg protein^−1^.

### 4.3. In Vitro Data Related to Distribution, and Metabolism

A value for plasma protein binding was retrieved from the US EPA Comptox platform [48]. Methods for measuring the blood/plasma ratio and metabolic stability (liver) measurements are described in Sections S1.2 and S1.3 of the Supplementary Materials, respectively. The protocol to measure the blood/plasma ratio was adapted from a method by Yu et al. [56].

Metabolism of oxybenzone in cryopreserved primary human hepatocytes was observed to be mainly due to demethylation and glucuronidation (See Supplementary Figures S2 and S3). Based on the findings of others [57], for highly protein-bound chemicals such as oxybenzone, the in vivo hepatic clearance is very likely to be significantly underestimated when the standard well-stirred equation includes corrections for the unbound fraction in plasma; therefore, the non-restrictive equation below was used to calculate liver clearance.

(5) $${CL}_{liver}=\frac{Q_{liver}\times in\;vivo\;CL_{int}\times Rbp}{Q_{liver}+in\;vivo\;CL_{int}}$$

where CL_liver_ is the hepatic plasma clearance, Q_liver_ is the hepatic blood flow (human, 90 L/h), CL_int_ is the intrinsic clearance (µL·min^−1^·mg protein^−1^), and Rbp is the blood/plasma concentration ratio.

### 4.4. Model Development

The workflow described in the OECD guidance 2021 [17] regarding the steps for development, validation, reporting, and discussion was followed. In silico parameters and ECCS class [58] were predicted using ADMET predictor 10.3.0 (MW, solubility in water, pKa) and experimental measurements of in vitro skin parameters were conducted for the two formulations containing oxybenzone used in the clinical study [18,19]. In vitro ADME parameters for hepatic metabolism and blood/plasma ratio were generated as described above.

**Figure 6:**
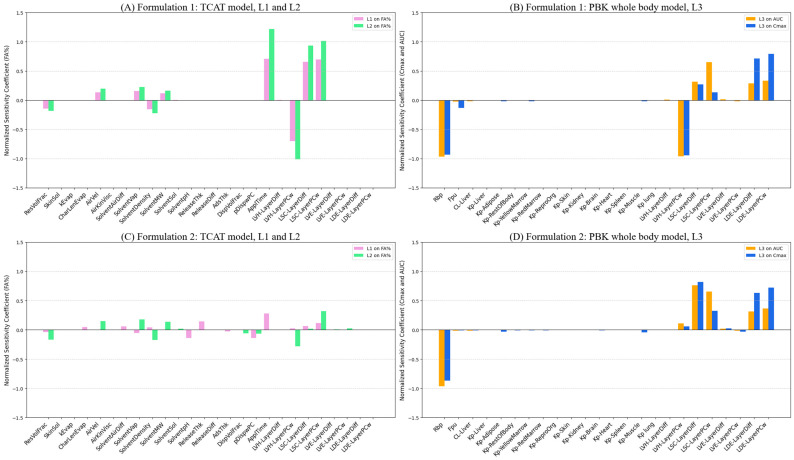
Parameter Sensitivity analysis for (**A**) Formulation 1 using the TCAT model at L1 and L2, (**B**) Formulation 1 using the PBK whole body model at L3, (**C**) Formulation 2 using the TCAT model at L1 and L2, (**D**) Formulation 2 using the PBK whole body model at L3. Fa%: absorbed fraction from the skin. In (**A**,**C**), Level 1 (L1) is denoted by pink bars and Level 2 (L2) is denoted by green bars. In (**B**,**D**), AUC is denoted by blue bars and C_max_ is denoted by orange bars. The sensitivity was classified as follows: high ≥ 0.5; medium < 0.5 and ≥0.2; low ≤ 0.2 and ≥0.1 [16].

The PBK model was built using GastroPlus 9.9 (Simulation Plus, Lancaster, CA, USA). First, a TCAT in vitro model was built, then the different input parameters were refined by optimization of the measured in vitro concentrations in the different layers of the skin according to the results of a sensitivity analysis (Figure 6). The resulting model was then combined with the full body model. The full body model was parametrized with additional in vitro parameters such as clearance in hepatocytes. This workflow was applied for both formulations containing oxybenzone. Finally, the predicted plasmatic concentrations versus time profiles after a single and repeated dermal application were compared to the respective clinical data for each of the two [18,19] as an external set to validate the models.

#### 4.4.1. Dermal Model

The TCAT module from GastroPlus 9.9 was used to develop the dermal module of the PBK model. In the L1 approach, only default and in silico-predicted parameters were used. In the L2 approach, in vitro skin penetration (concentrations in skin layers (see Supplementary Figure S1B), partition and diffusion coefficients, thickness of the different skin layers), kinetics in isolated dermis, and % residual formulation after evaporation data were used to parameterize the in vitro TCAT models and optimized using the in vitro skin absorption data. If no measured value was available, then predicted values were used (e.g., Sw, pKa) to complete parameters required by TCAT (Supplementary Table S5b).

The vehicle effect was included in the model by using the diffusivity and partitioning of the specific vehicle. The formulation type for Formulation 1 was set to “solution” and “lotion” for Formulation 2. In the settings, the solvent was set as “volatile”. The evaporation rate of the vehicle was calculated using the Peress equation [54]. While oxybenzone is not soluble in water, it is solubilized in the formulations; therefore, the solubility value used in TCAT model corresponds to the concentration of oxybenzone in the formulations. Considering the low to negligible metabolism in skin S9, the contribution of skin metabolism to the changes in the chemical amount in the skin was ignored (see Section 4.2.5 Skin metabolism data).

As described above, except for D_VE_ and D_VH_, all other values were generated experimentally. Diffusivity in the viable epidermis (D_VE_) was derived from the optimized equation (Robinson equation [26]). Diffusivity in the vehicle (D_VH_) was taken to be 7.5 × 10^−6^ cm^2^·s^−1^, which is the value provided by GastroPlus 9.9 for aqueous vehicles. For the dermis, the measured diffusivity value on isolated dermis was used, D_DE_ = 1.27 × 10^−7^ cm^2^·s^−1^.

Experimental data for the thickness of stratum corneum, epidermis, and dermis were used as input. The thickness of the donor compartment (vehicle) was calculated by the software. The volume of distribution was matched to the receiver chamber in Franz cell (Nebo, Les Lilas, France) (3 mL).

The contribution of sebum is negligible in in vitro skin absorption assays; therefore, when scaled up to in vivo situations, the absorption was calculated without accounting for sebum.

Data from the in vitro skin absorption assay for the four formulations were used to optimize chemical-specific parameters of the skin; however, the full TCAT models were only built for Formulations 1 and 2. Of note, for both L1 and L2 models, chemical-specific parameters were optimized using all four formulations, and formulation-specific effects were optimized using Formulation 1 and Formulation 2 skin absorption data.

#### 4.4.2. Refinement of Chemical-Specific Parameters

For the optimization of the TCAT model, it was assumed that some of the parameters, such as D_VE_, K_VE/w_, D_DE_, and K_DE/w_, are related to the intrinsic physicochemical characteristics of oxybenzone and not to the composition of the formulations. Therefore, the first step was to simultaneously optimize the parameters related to the chemical for all the four tested formulations, using the kinetics obtained from skin absorption after 4, 16 and 24 h. The software option “All records optimization” was used for this purpose.

#### 4.4.3. Refinement of Formulation-Specific Parameters

In the second step, the parameters, K_VH/w_, D_STCOR_, K_STCOR/w_ and application time, were optimized separately for Formulation 1 and 2. D_VH_ was not optimized because it did not appear to be a sensitive parameter in the PSA performed before the optimization. When optimizing such parameters, the maximum and minimum reported in the literature were used to limit the sampling range for the algorithm. Here, a weight of 1 to the stratum corneum, as well as to the epidermis, dermis and receptor fluid was used. The GastroPlus 9.9 “help document”: optimization module from GastroPlus 9.9) defines the “objective function” as follows: The error term based on the differences between predicted and observed values for any combination of skin layer concentration–time. The different available objective functions proposed by the software (1/Y^2^, 1/Ỹ^2^ and 1/(Y + Ỹ)^2^) were tested and the latter which resulted in the lowest value for the objective function and a profile consistent with the concentration–time kinetics in the receptor fluid was selected (see Supplementary Table S5).

#### 4.4.4. Human Full Body PBK Model

##### Application Scenarios

The PBK model was based on the single dose application scenario as reported in Dataset A, based on the clinical report [19], as described in the Materials and Methods. In this study, two US commercially available sunscreen formulations, Formulation 1 and 2, containing oxybenzone and other UV filters under maximal use conditions were topically applied to 12 human volunteers.

For the in vivo TCAT module, the site of exposure was selected as “Human abdomen”, which represents the largest surface area among the options provided by the software. Of note, 75% of the body was exposed to the sunscreen in the clinical trial. For the “single dose”, the applied quantity for Formulations 1 and 2 were 1710 and 1140 mg, respectively, which were applied in 28.5 mL formulation to 14,250 cm^2^ of skin. The sunscreens were applied once a day. Application on the skin was set as non-occlusive.

##### PBK Model Parameterization

Supplementary Table S5 lists the values used for parametrization of the PBK model. A measured value of 0.693 for the Rbp was used as input. The free fraction in plasma of 1% from the US EPA database [48] was used as input.

ADMET predictor 10.3.0 was used for the ECCS classification. Oxybenzone was flagged to be eliminated mainly via liver clearance (Class 2, high permeable, Neutrals/Bases). Hence, renal transporters were not considered when parametrizing the model. The renal clearance was set to Fup × glomerular filtration rate mL/s [31]. All compartments/tissues were assumed to be perfusion limited. The GastroPlus 9.9 method Lukacova/Rogers-Single [59] for tissue/plasma partition coefficient (Kp) calculation was used. The measured in vitro intrinsic clearance (CL_int_) in hepatocytes was converted to the in vivo intrinsic CL value as described above. The systemic absorption rate constant (ksys) was estimated using the Ibrahim integrated model [28]. The individual characteristics were specified based on those of the volunteers in the clinical study, i.e., American male, with age and weight values as follows: for Formulation 1, 41 years old, 76 kg, for Formulation 2, 45 years old, 76 kg. GastroPlus 9.9 default settings were used for the number of human compartments.

#### 4.4.5. PBK Model Performance

##### Sensitivity Analysis

The sensitivity analysis provides a quantitative evaluation of how input parameters influence the dose metric simulations. This was calculated using the Parameter Sensitivity Analysis (PSA) analysis function in GastroPlus 9.9 with a variation of ±5% according to Evans and Andersen [60]) on the output parameters (C_max_ and/or AUC) for the Rbp, Fup, in vitro CL in liver, D_VH_, K_VH/w_, D_STCOR_, K_STCOR/w_, D_VE_, K_VE/W_, D_DE_, K_DE/W_, Application time, Residual volume fraction, and Evaporation rate. This is carried out by adding a perturbation to one parameter (x) at a time while keeping all the other parameters constant [61]. The analysis involved calculating a sensitivity coefficient (S) normalized by the input and output of the model according to Equation (6):

(6) $$S=\left|\frac{\Delta y}{\Delta x}\frac{x}{y}\right|$$

where y is the output data (e.g., C_max_, T_max_, AUC), x is the input parameter (Rbp, Fup, CL in liver, D_STCOR_, K_STCOR/w_, D_VH_, K_VH/w_…), Δx is the change in input parameters, and Δy is the change in output parameters.

A sensitivity ratio of 1 implies that a 5% change in the input of a parameter value results in a 5% change in the AUC. The sensitivity was classified as follows: high ≥ 0.5; medium < 0.5 and ≥0.2; low ≤ 0.2 and ≥0.1 [16].

##### Uncertainty Analysis

Uncertainty analysis evaluates the impact of the lack of precise knowledge of parameter values and model structure on dose metric simulations. This was conducted according to the WHO guidelines [16] using the Population Estimates for Age Related Physiology (PEAR) module in GastroPlus 9.9. The uncertainty is expressed as a %CV, which was derived from experimental values or from data within the Gastroplus 9.9 software. The uncertainty for a population simulation of 100 subjects was calculated by varying multiple parameters with a relative CV%, for a Log-Normal distribution (see Supplementary Tables S6–S9). Values of the 2.5th, 50th and 97.5th percentiles were generated for the C_max_ and/or AUC [62]. Uncertainty was determined by the values of C_max_ at 97.5th percentile minus 2.5th percentile over those for the 50th percentile. The resulting uncertainty values were classified as follows: high ≥ 2; medium < 2 and ≥0.3; low ≤ 0.3 [16].

##### Reliability

The reliability of a PBK model used for risk assessment depends essentially on its ability to provide reliable predictions of the dose metric. According to the WHO [16] guideline, reliability is determined from the results of the sensitivity and uncertainty analysis performed. As mentioned above, sensitivity and uncertainty are each classified into three levels of high, medium and low. If a PBK parameter is highly sensitive and highly uncertain, the model’s reliability is rated as low.

##### Simulation for Human Population

The PEAR module was used to evaluate the variability of the C_max_ or AUC_(0–t)_ values within an example population. The built-in GastroPlus 9.9 algorithm was used to account for sex, age, and body weight-dependent changes in the physiological and anatomical parameters, such as blood flow, cardiac output, and organ/tissue volumes. For Formulations 1 and 2, the simulation was performed for a population of 500 subjects. These were healthy American subjects (the European population is not available in the software), 50% men and 50% women, between 18 and 70 years of age, and a weight between 50 and 90 kg. Within this population analysis, variability of in silico/in vitro parameters were also considered. The model was run including the related CV% for a Log-Normal distribution. The outputs of the Population Simulator are mean values (arithmetic and geometric), coefficient of variation (CV) and confidence intervals of 90% for C_max_, T_max_ and AUC. The in vitro/in silico input data of the PK parameters are listed in Supplementary Table S5b and the value ranges of both the physiological population parameters are listed in Table 3.

##### PBK Model External Validation

In PBK modelling, predictions that are, on average, within a factor of 2 of the experimental data have frequently been considered adequate [16]. The mean plasma concentrations measured in 12 subjects per formulation were used to compare with the simulated values. The details of the PK parameters and subjects for the simulations are listed in Supplementary Table S5 and Table 3, respectively. PK metrics resulting from a single dose application of Formulations 1 and 2 were compared to the clinical data in Dataset A [18]. Additionally, multiple dose application plasma concentrations in Dataset A were also used to assess the model’s performance. For the “multiple dose” simulations, the applied quantity and the surface area used as input for Formulation 1 and 2, were 1710 and 1140 mg, respectively. These were applied in 28.5 mL formulation to 14,250 cm^2^ of skin. The sunscreens were applied once on Day 1 (corresponding to the “simple dose”) and then four times (with 2 h intervals) on Day 2 through Day 4, to 75% of the body surface area. Of note, multiple-dose simulations were continued long enough to establish steady-state concentrations of the substance.

## 5. Conclusions

In conclusion, in this study, PBK models were built and refined using only in silico (L1), in silico and in vitro (L2), and refined using clinical (L3) data to predict the PK profile of oxybenzone present in two commercial sunscreen formulations under maximal use conditions. An evaluation showed that there was a high confidence in the L2 models which was comparable with the L3 simulations. L2 models could describe the effect of formulation type, indicating they could be used in similar cases where differences are observed in the absorption across two different formulation types. The construction of the PBK model of oxybenzone differed from the current available literature for similar UV filters, particularly the approach for a formulation-dependent cutaneous parameterization in two stages: (1) chemical-specific parameters (where an average of the obtained values across different formulations or an optimized value using data from absorption of several formulations containing the same sunscreen is used), (2) formulation-specific parameters using two independent formulations in lotion (hydrophilic) and spray (hydrophobic).

The optimization steps are very important for chemicals like oxybenzone because the plasma C_max_ is driven by the chemical’s dermal permeability and Rbp. Understanding the necessary experiments for generating input parameters is crucial and raises several pertinent questions. For instance, the need for measured distributions across the skin at various times (kinetics), and whether measured partition coefficients are more accurately represented using alternative methods, as suggested by Ellison et al. [22]. Furthermore, effective management of the constant systemic absorption rate (ksys) and application time within the GastroPlus 9.9 software requires further investigation. When clinical PK data are unavailable, which is frequently the scenario for cosmetic ingredients, the need for robust dermal input parameters becomes paramount to ensure the highest level of reliability. This underscores the significance of establishing clear guidelines for conducting skin absorption studies that are specifically tailored to yield valuable data inputs for the development of PBK models.

The results of this study demonstrate how formulation-dependent absorption kinetics improve confidence in safety assessments, aligning with regulatory demands for animal-free testing paradigms and thereby ensuring its potential use in the context of NGRA.

## Figures and Tables

**Table 1:** Predicted and measured PK values of oxybenzone after single and repeated application of oxybenzone in (**A**) Formulation 1 and (**B**) Formulation 2. The ratio of predicted to observed, for C_max_ and AUC, for single dose application, are calculated on predictions for a population of 2500 people. When assessing multiple dose application, one subject was considered. * Since the simulated data did not reach a C_max_, the extrapolated AUC cannot be calculated.

(**A**) **Formulation 1**					
Dosing Schedule	Output parameter	Observed	Predicted L1	Predicted L2	Predicted L3
Single dose 1 app/day (Day 1) (1710 in 28.5 mL)	C_max_ (µg/mL)	0.0894	0.06524	0.0633	0.0627
	T_max_ (h)	9.00	23	5.75	6.82
	AUC_0–∞_ (µg·h/mL)	2.37	NA *	0.97294	9.69
	AUC_0–23_ (µg·h/mL)	1.29	0.96534	0.8147	1.30
	C_max Pred/Obs_ ratio		0.729754	0.708054	0.701
	AUC_0–23 Prd/Obs_ ratio		0.746	0.630	1.01
	CL_sys_(L/h)		5.299	64.669	27.319
	V_ss_ (L)		400.327	138.201	82.008
Sim Pop 1 app/day (Day 1) (1710 in 28.5 mL)	Number of subjects		-	500	500
	C_max_ (µg/mL) mean		-	0.0982	0.063
	CV%		-	150.99	59.8
	min		-	0.00224	0.00698
	max		-	1.6827	0.231
	CI 90%		-	0.087 to 0.109	0.060 to 0.0650
	AUC_0–23_ (µg·h/mL)			1.0526	1.28
	CV%			114.05	58.0
	min			0.03435	0.150
	max			10.824	4.56
	CI 90%			0.964 to 1.141	1.22 to 1.33
Multiple dose 1 app/day 1 4 app/day 2-3-4 2 h of interval (1710 in 28.5 mL)	C_max_ (µg/mL)	0.170	-	0.278	0.134
	T_max_ (h)	73.0	-	79.2	80.4
	AUC_0–∞_ (µg·h/mL)	16.4	-	14.6	28.4
	AUC_0–23_ (µg·h/mL)	15.6	-	14.6	27.1
	C_max Pred/Obs_ ratio			1.64	0.788
	AUC_0–t Prd/Obs_ ratio			0.936	1.74
(**B**) **Formulation 2**					
Dosing Schedule	Output parameter	Observed	Predicted L1	Predicted L2	Predicted L3
Single dose 1 app/day (Day 1) (1140 in 28.5 mL)	C_max_ (µg/mL)	0.0800	0.05082	0.047	0.084
	T_max_ (h)	6.00	23	5.6733	3.760
	AUC_0–∞_ (µg·h/mL)	1.89	NA *	0.70546	1.820
	AUC_0–23_ (µg·h/mL)	1.26	0.75961	0.5965	1.270
	C_max Pred/Obs_ ratio		0.632877	0.585	1.05
	AUC_0–23 Prd/Obs_ ratio		0.603	0.473	1.01
	CL_sys_(L/h)		5.299	64.669	59.570
	V_ss_ (L)		400.327	138.201	81.683
Sim Popm 1 app/day (Day 1) (1140 in 28.5 mL)	Number of subjects		-	500	500
	C_max_ (µg/mL) mean		-	0.07788	0.084
	CV%		-	153.79	72.500
	min		-	0.0009136	0.00657
	max		-	1.5748	0.381
	CI 90%		-	0.069 to 0.087	0.0800 to 0.0890
	AUC_0–23_ (µg·h/mL)			0.81645	1.26
	CV%			115.56	66.7
	min			0.009218	0.129
	max			9.3286	5.28
	CI 90%			0.747 to 0.886	1.20 to 1.32
Multiple dose 1 app/day 1 4 app/day 2-3-4 2 h of interval (1140 mg in 28.5 mL)	C_max_ (µg/mL)	0.210	-	0.169	0.128
	T_max_ (h)	78.0	-	82.1	79.7
	AUC_0–∞_ (µg·h/mL)	17.3	-	9.02	9.2
	AUC_0–23_ (µg·h/mL)	16.8	-	9.02	9.2
	C_max Pred/Obs_ ratio			0.805	0.610
	AUC_0–23 Prd/Obs_ ratio			0.537	0.549

**Table 2:** Reliability of the whole body PBK model for Formulation 1 (A) and Formulation 2 (B) based on the sensitivity and uncertainty analyses with parametrization with in silico and in vitro data (L2). Background color: darkest grey indicates low reliability, medium grey indicates medium reliability, light grey indicates high reliability.

		(**A**) **Formulation 1**		
		**Uncertainty in variability of the input parameter estimates**		
		High	Medium	Low
**SENSITIVITY**	High	**K_VH/w_**	**Rbp**	
	Medium		**D_STCOR_, D_DE_, K_DE/w_**	
	Low		**K_STCOR/w_**	**Fup, D_VH_, D_VE_, K_VE/w_, Solvent Residual Volume Fraction, K_evap_**
		(**B**) **Formulation 2**		
		**Uncertainty in variability of the input parameter estimates**		
		High	Medium	Low
**SENSITIVITY**	High	**D_STCOR_**	**Rbp, K_VH/w_, K_STCOR/w_, D_DE,_ K_DE/w_**	
	Medium			
	Low		**Fup, D_VH_, K_VE/w_, D_VE,_ Solvent Residual Volume Fraction, K_evap_**	

**Table 3:** Population parameters and corresponding values used for in vivo simulations of the plasma concentrations in a single subject who applied a formulation containing oxybenzone. Formulation 1 = Aerosol spray-Neutrogena SPF100; Formulation 2 = Lotion Hawaiian tropic. Values were from Matta et al. (2020) [18].

PBK Model	Formulation 1	Formulation 2
Species	Human	Human
Population	American	American
Gender	Male	Male
Age (year)	41	45
Weight (kg)	76	76,3
Height (cm)	176.23	176.01
Dose (mg)	1710	1140
Volume (ml)	28.5	28.5
Application surface area (cm^2^)	14,250	14,250

## Data Availability

The original contributions presented in this study are included in the article/Supplementary Material. Further inquiries can be directed to the corresponding author.

## Supplementary Materials

The following supporting information can be downloaded at: https://www.mdpi.com/article/10.3390/ph18111607/s1.

## Abbreviations

absorption, distribution, metabolism and excretion (ADME); blood/plasma ratio (Rbp); Coefficient of Variation (CV); Parameter Sensitivity Analysis (PSA); Fraction unbound from plasma proteins (Fup); Rapid Equilibrium Dialysis (RED), Diffusivity in the dermis (D_DE_); Diffusivity in the viable epidermis (D_VE_); diffusivity in the skin (D_skin_); diffusivity in the stratum corneum (D_STCOR_); diffusivity in the vehicle (D_VH_); European Union (EU); margin of safety (MoS); Organization for Economic Co-operation and Development (OECD); Partition coefficient between the dermis and water (K_DE/w_); Partition coefficient between the viable epidermis and water (K_VE/w_); Partition coefficient between the receptor fluid and water (K_RF/w_); Partition coefficient in the stratum corneum and water (K_STCOR/w_); Partition coefficient between the vehicle and water (K_VH/w_); Systemic absorption rate (k_sys_); Physiologically based kinetics (PBK); Scientific Committee on Consumer Safety (SCCS); tissue/plasma partition coefficient (Kp); transepidermal water loss (TEWL); World Health Organization (WHO).
