# Supplementary material for: A Novel Workflow for Non-Animal PBK Modelling of UV Filters: Oxybenzone as a Case Study
Source: Pharmaceuticals (Basel). 2025 Oct 24;18(11):1607. doi: 10.3390/ph18111607 (PMC12655505; doi:10.3390/ph18111607)
Supplement: Supplementary file 1 [file pharmaceuticals-18-01607-s001.zip › pharmaceuticals-3805926-supplementary.pdf]

Supplementary Materials

1 Supplementary Materials and Methods

1.1 Oxybenzone Analytical method

Supplementary Table S1: HPLC information

Device	Binary Pump	Acquity	K10UPB205A		
	Auto-Sampler	Acquity	K10UPA091M		
	Oven with column	Acquity	C10UPH550G		
	TUV Detector	Acquity	H10TUV256A		
	Photo-diode Array Detector	Acquity PDA eλ	L10UPL705A		
Column	Acquity UPLC BEH C18 1.7µm 2.1x100 mm				
Solvent A	Trifluoroacetic acid 0.1% in water				
Solvent B	Acetonitrile				
Temperature of the oven (°C)	50				
Weak solvant	Acetonitrile/water (10/90 v/v)				
Strong Solvant	Acetonitrile				
Injection Solvent	Methanol/DMSO (50/50 v/v) or PBS 0.1M + 4% Brij O20				
Injection Volume (µL)	5 µl for washing, ST, Epidermis, Dermis<br><br>10 µl for LR				
**Gradient**	Time (min)	Flow (ml/min)	A (%)	B (%)	Curve
	0	0.6	70	30	Initial
	5	0.6	0	100	5
	7	0.6	0	100	6
	7.1	0.6	70	30	6
	10	0.6	70	30	6

- **At 10µl, DMSO peak disturbs the integration of Oxybenzone peak and the oxybenzone peak is doubled.*

Supplementary Table S2: Retention time and maximum wavelength used for quantification

	RT (min)	λ (nm)
Oxybenzone	1.96	322

Supplementary Table S3: Lowest limit of quantification and limit of detection for each chemical using LC/UV method

		Limit of Detection (ng/ml)		Lowest Limit of Quantification (ng/ml)	
Compartments		Washing<br>Strips<br>Epidermis<br>Dermis	RF	Washing<br>Strips<br>Epidermis<br>Dermis	RF
Filter	Oxybenzone	9.8	9.8	9.8	19.5

Typical chromatogram (322nm) at 1250 ng/ml in DMSO/MeOH

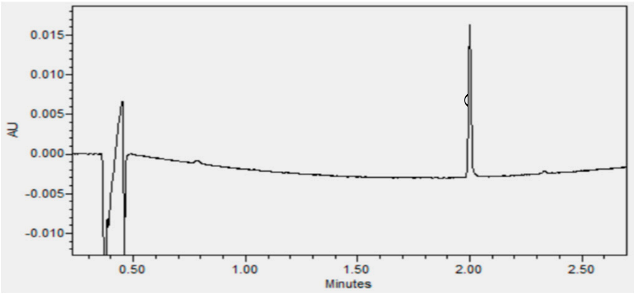

1.2 Measurement of the Blood:plasma Ratio

The blood:plasma ratio was measured by Cyprotex (UK). The protocol was adapted from a method by Yu et al. (2005). Oxybenzone (in triplicate) was incubated at 0.5 µM with human whole blood for 60 min at 37 °C. Following the incubation, the whole blood samples were centrifuged for 5 min at 5,000 xg at 4°C. An aliquot was sampled from the plasma and red blood cell layers for analysis. Red blood cell layer was freeze thawed quickly three times to lyse the red blood cells.

A separate aliquot of reference blood was centrifuged, and the red blood cell portion and the plasma portion were spiked separately with test compound and incubated alongside the whole blood. After protein precipitation and centrifugation, the supernatants for the experimental samples and reference samples were analysed by LCMS/MS.

1.3 Liver metabolism assays (hepatocyte)

US-EPA measured *in vitro* intrinsic hepatic clearance on hepatocytes at 15.7 µl/min/million hepatocytes. The study was done based on the OECD TG 319A guideline for trout hepatocytes. The evaluation was carried out using cryopreserved primary human hepatocytes (PPH). Chemical was incubated at 5 µM over 120 min in

triplicate at 37°C. The concentration of PHH was 1.0 x 106 cells. ml-1. Sampling points were made at 0, 15, 30, 60, 90 and 120 min. Samples were quantified by LC/MS (See **Supplementary Section 1.1**).

The metabolic activity of hepatocytes was confirmed by incubating 7-ethoxycoumarin. A half-life of 0.14 ± 0.06 h was obtained for this reference control, indicative of a good metabolic capability of these hepatocytes.

1.4 Gastroplus software input for the TCAT model; calculation of skin layers diffusivity and partition coefficient

GastroPlus software can incorporate input values for the exact thickness of the skin layers used in the experiments. The measurements of stratum corneum thickness are shown in Supplementary Table S4.

Supplementary Table S4: Measured stratum corneum thicknesses of the human skin used in the skin absorption experiments. These were used as input for the TCAT in vitro module.

Donor number	Age	Dermis thickness (µm)	Epidermis (µm – mean)	Stratum corneum thickness (µm – mean ± SD)
1	50	46.95	751.14	13.91 ± 1.11
2	66	53.85	745.39	12.76 ± 0.70
3	61	70.30	687.58	14.12 ± 1.65
4	61	53.30	677.68	13.02 ± 1.69
5	34	46.80	630.32	13.88 ± 1.68
6	-	60.55	650.68	10.77 ± 1.99
7	30	65.70	626.73	12.57 ± 1.42
8	32	58.95	635.34	12.71 ± 1.11
9	33	55.90	954.24	12.86 ± 1.09
10	48	40.45	835.24	10.31 ± 0.74
11	71	59.30	968.33	12.37 ± 1.12
12	41	79.65	807.45	12.90 ± 0.89
13	53	50.35	1306.63	13.02 ± 1.38
14	63	51.30	984.33	10.37 ± 1.11
15	24	64.75	802.88	14.37 ± 1.27
16	57	61.00	901.29	11.71 ± 0.66

GastroPlus software uses water partition coefficients (K_w). Thus, experiment partition coefficients ($K_{STCOR/VH}$, $K_{VE/STCOR}$, $K_{VE/DE}$ and $K_{RF/DE}$) were converted to water partition coefficients ($K_{VH/W}$, $K_{STCOR/W}$, $K_{VE/W}$ and $K_{DE/W}$) using the following equations:

Equation 1 $$K_{\frac{VE}{VH}} = K_{\frac{STCOR}{VH}} \times K_{\frac{VE}{STCOR}}$$

Equation 2 $$K_{\frac{DE}{VH}} = K_{\frac{DE}{VE}} \times K_{\frac{VE}{VH}}$$

The RF used in this study was phosphate buffer solution containing 4% Brij O20. This surfactant was added to ensure oxybenzone's solubility and sink conditions. Despite presence of the surfactant, it was assumed that the value for $K_{RF/w}$ was equal to 1:

Equation 3 $$K_{\frac{RF}{DE}} = K_{\frac{W}{DE}} = \frac{1}{K_{\frac{DE}{W}}}$$

Based on the $K_{DE/W}$ and $K_{DE/VH}$ the partitioning between the vehicle and water was calculated as follows:

Equation 4 $$K_{\frac{VH}{W}} = \frac{K_{\frac{DE}{W}}}{K_{\frac{DE}{VH}}}$$

This value was used to calculate the partition coefficients between stratum corneum and viable epidermis with water:

Equation 5 $$K_{\frac{STCOR}{W}} = K_{\frac{STCOR}{VH}} \times K_{\frac{VH}{W}}$$

Equation 6 $$K_{\frac{VE}{W}} = K_{\frac{VE}{VH}} \times K_{\frac{VH}{W}}$$

Where K is the partition coefficient, VE is the viable epidermis, VH is the vehicle, STCOR is the stratum corneum, RF is the receptor fluid, DE is the Dermis, and W is water.

1.5 Values used to parametrize the PBK model for the simulations

Supplementary Table S5. (A) Modules for calculation and (B) Parameters and corresponding values used for the simulations of oxybenzone in: Formulation 1 = Aerosol spray; Formulation 2 = Lotion. The value used for the ksys parameter was not directly accessible through the software interface but was instead obtained by contacting the developers of the TCAT model (ksys = 1.07 x 10^{-3} S^{-1} [28]).

(a) Modules for calculation.

PBK module	Equation used	Sources
Tissue model	Perfusion limited tissues	GastroPlus
Calculation of Kps perf Kp	Lukacova (Rogers-single)	GastroPlus
Fut method	S+v9.5	GastroPlus
Ksys (dermis to systemic rate constant)	Ibrahim	GastroPlus
Vehicle evaporation rate constant model	Peress	GastroPlus

(b) Parameters and corresponding values used for the simulations of oxybenzone.

Parameter	Value	Source	PBK level
MW (g/mol)	228.2	ADMET predictor	L1, L2, L3
Log P	3.61	ADMET predictor	L1
	3.38	Measured, Median of two experimental values (US-EPA)	L2, L3
pKa	10.1 (AH/A-)	ADMET predictor	L1, L2, L3
Water solubility (mg/ml)	0.071	ADMET predictor	L1
	0.006 (25°C)	Measured (ECHA)	L2, L3
Fraction unbound in plasma (Fup)	0.0536	ADMET predictor	L1
	0.01	Measured by USEPA	L2, L3
Blood: plasma ratio	0.879	ADMET predictor	L1
	0.693	Measured	L2, L3
Intrinsic clearance on hepatocytes (µl/min/10^6) with Fu hep assumed to be 0.01	167	ADMET predictor	L1
	295	Measured (pooled human liver hepatocytes)	L2, L3
Permeability effective Peff (cm/s)	9.411	ADMET predictor	L1
Renal Cl_{sys} (l/h)	0.06667	GFR*Fup	L1, L2, L3
Experimental vehicle residual volume	54.9	Measured for Formulation 1	L1, L2, L3
	27.5	Measured for Formulation 2	L1, L2, L3
Solvent evaporation rate (ml/s)	0.82843	Calculated for Formulation 1	L1, L2, L3
	0. 16657	Calculated for Formulation 2	L1, L2, L3
Application time (h)	5.282E-05	Fitted against skin penetration data for Formulation 1	L1
	7.928E-05	Fitted against skin penetration data for Formulation 2	L1
	0.00431	Calculated	L1, L2 (Formulation 1)
	0.0345	Calculated	L1, L2 (Lotion)
	1.222×10^{-2}	Fitted against skin penetration data for Formulation 1	L2
	3.523×10^{-2}	Fitted against skin penetration data for Formulation 2	L2
	0.0001065	Fitted against clinical data for Formulation 1	L3
	0.00099	Fitted against clinical data for Formulation 2	L3
Vehicle/water diffusivity (cm^2/s)	7.5×10^{-6}	Calculated	L1, L2
	1.10×10^{-4}	Fitted against clinical data for Formulation 1	L3
	7.746×10^{-4}	Fitted against clinical data for Formulation 2	L3
Vehicle/water partition coefficient	1	GastroPlus default value	L1
	1.101	Fitted against skin penetration data for Formulation 1	L1
	5.179×10^{5}	Fitted against skin penetration data for Formulation 1	L2

	30108	Fitted against clinical data for Formulation 1	L3
	1.534	Fitted against skin penetration data for Formulation 2	L1
	9.412×10^{5}	Fitted against skin penetration data for Formulation 2	L2
	18395	Fitted against clinical data for Formulation 2	L3
Stratum corneum/water partition coefficient	55	Wang-Kasting-Nitsche method	L1
	3.985	Fitted against skin penetration data for Formulation 1	L1
	2.044×10^{6}	Fitted against skin penetration data for Formulation 1	L2
	33494	Fitted against clinical data for Formulation 1	L3
	3.337	Fitted against skin penetration data for Formulation 2	L1
	1.351×10^{6}	Fitted against skin penetration data for Formulation 2	L2
	3201	Fitted against clinical data for Formulation 2	L3
Stratum corneum diffusivity (cm^2/s)	4.86×10^{-11}	Wang-Kasting-Nitsche method	L1, L2 (Lotion)
	8.367×10^{-9}	Fitted against skin penetration data for Formulation 1	L1
	1.744×10^{-11}	Fitted against skin penetration data for Formulation 1	L2
	1.5×10^{-6}	Fitted against clinical data for Formulation 1	L3
	1.009×10^{-8}	Fitted against skin penetration data for Formulation 2	L1
	1.772×10^{-11}	Fitted against skin penetration data for Formulation 2	L2
	7.83×10^{-7}	Fitted against clinical data for Formulation 2	L3
Epidermis/water partition coefficient	1	Robinson method	L1
	0.125	Fitted against skin penetration data for Formulation 1 and 2	L1
	62.78	Measured in *ex vivo* human skin	L2
	98138.25	Fitted against skin penetration data for Formulation 1 and 2	L2
	291	Fitted against clinical data for Formulation 1 and 2	L3
Epidermis diffusivity (cm^2/s)	2.64×10^{-7}	Robinson method	L1, L2
	8.162×10^{-5}	Fitted against skin penetration data for Formulation 1 and 2	L1
	8.439	Fitted against skin penetration data for Formulation 1 and 2	L2
	4.21×10^{-9}	Fitted against clinical data for Formulation 1 and 2	L3
Dermis/water partition coefficient	0.7	Robinson method	L1
	15.75	Measured in *ex vivo* human skin	L2
	2.032×10^{-2}	Fitted against skin penetration data for Formulation 1 and 2	L1

	10884.347	Fitted against skin penetration data for Formulation 1 and 2	L2
	16.7	Fitted against clinical data for Formulation 1 and 2	L3
Dermis diffusivity (cm^2/s)	2.02×10^{-6}	Robinson method	L1
	1.27×10^{-7}	Measured in *ex vivo* human skin	L2
	1.744×10^{-8}	Fitted against skin penetration data for Formulation 1 and 2	L1
	1.682×10^{-3}	Fitted against skin penetration data for Formulation 1 and 2	L2
	1.76×10^{-7}	Fitted against clinical data for Formulation 1 and 2	L3
% Oxybenzone in formulation	6	Formulation 1: Aerosol Spray (Matta et al.)	L1, L2, L3
	4	Formulation 2: Lotion (Matta et al.)	L1, L2, L3
Dose (mg)	0.24	Formulation 1: Aerosol Spray (Matta et al.)	L1, L2, L3
	0.16	Formulation 2: Lotion (Matta et al.)	L1, L2, L3
Dose volume (ml)	0.004	Formulation 1: Aerosol Spray (Matta et al.)	L1, L2, L3
	0.004	Formulation 2: Lotion (Matta et al.)	L1, L2, L3

1.6 Parameters used for global population *in vivo* simulations for two formulations at level L2 and L3

Supplementary Table S6: Formulation 1: L2

Parameter	Lower Limit	Mean value	Upper Limit	CV%	Distribution
blood plasma	0.678	0.693	0.708	0.73	Log-Normal
fraction unbound	0.658	1.00	1.52	15	Log-Normal
$K_{VH/w}$	1.2×10^5	5.18×10^5	2.24×10^6	62.9	Log-Normal
$K_{STCOR/w}$	4.73×10^5	2.05×10^6	8.84×10^6	62.9	Log-Normal
$K_{VE/w}$	2.27×10^4	9.81×10^4	4.24×10^5	62.9	Log-Normal
$K_{DE/w}$	2517.9	1.09×10^4	4.71×10^4	62.9	Log-Normal
D_{VH}	5.63×10^{-6}	7.50×10^{-6}	9.98×10^{-6}	10	Log-Normal
D_{STCOR}	4.034×10^{-12}	1.74×10^{-11}	7.54×10^{-11}	62.9	Log-Normal
D_{VE}	6.34	8.44	10	10	Log-Normal
D_{DE}	3.89×10^{-4}	0.0017	0.0073	62.9	Log-Normal
Application Time *in vivo*	0.0079	0.0122	0.019	15.8	Log-Normal
Solvent Residual Volume fraction	0.464	0.549	0.650	5.8	Log-Normal
CLint liver	4.89×10^4	3.63×10^5	2.70×10^6	95.1	Log-Normal
All other parameters				GP value	Log-Normal/Pear

Supplementary Table S7: Formulation 1: L3

Parameter	Lower Limit	Mean value	Upper Limit	CV%	Distribution
blood plasma	0.678	0.693	0.7083	0.73	Log-Normal
fraction unbound	0.6575	1	1.5209	15	Log-Normal
$K_{VH/w}$	6965	30108	130000	62.9	Log-Normal
$K_{STCOR/w}$	7747	33494	145000	62.9	Log-Normal
$K_{VE/w}$	67.4	291	1259	62.9	Log-Normal
$K_{DE/w}$	3.86	16.7	72.2	62.9	Log-Normal
D_{VH}	8.29×10^{-5}	1.10×10^{-4}	1.47×10^{-4}	10	Log-Normal
D_{STCOR}	3.66×10^{-7}	1.58×10^{-6}	6.83×10^{-6}	62.9	Log-Normal
D_{VE}	3.17×10^{-9}	4.21×10^{-9}	5.61×10^{-9}	10	Log-Normal
D_{DE}	4.07×10^{-8}	1.76×10^{-7}	7.60×10^{-7}	62.9	Log-Normal
Application Time *in vivo*	0.0000686	0.0001065	0.0001650	15.8	Log-Normal
Solvent Residual Volume fraction	0.4636	0.549	0.6502	5.8	Log-Normal
Cl liver	4.89×10^{4}	3.63×10^{5}	2.70×10^{6}	95.1	Log-Normal
All other parameters				GP value	Log-Normal/Pear

Supplementary Table S8: Formulation 2: L2

Parameter	Lower Limit	Mean value	Upper Limit	CV%	Distribution
blood plasma	0.678	0.693	0.708	0.73	Log-Normal
fraction unbound	0.658	1.00	1.52	15	Log-Normal
$K_{VH/w}$	1.97×10^{5}	9.41×10^{5}	4.5×10^{6}	68.5	Log-Normal
$K_{STCOR/w}$	1.02×10^{6}	1.35×10^{6}	1.8×10^{6}	68.5	Log-Normal
$K_{VE/w}$	2.05×10^{4}	9.81×10^{4}	4.7×10^{5}	68.5	Log-Normal
$K_{DE/w}$	2275.1	1.09×10^{4}	5.21×10^{4}	68.5	Log-Normal
D_{VH}	5.64×10^{-6}	7.50×10^{-6}	9.98×10^{-6}	10	Log-Normal
D_{STCOR}	3.704×10^{-12}	1.772×10^{-11}	8.477×10^{-11}	68.5	Log-Normal
D_{VE}	6.3404	8.4391	10	10	Log-Normal
D_{DE}	3.52×10^{-4}	0.0017	0.008	68.5	Log-Normal
Application Time *in vivo*	0.0216	0.0345	0.0549	16.8	Log-Normal
Solvent Residual Volume fraction	0.226	0.275	0.335	6.8	Log-Normal
Cl liver	5.28×10^{4}	3.92×10^{5}	2.91×10^{6}	95.1	Log-Normal
All other parameters				GP value	Log-Normal/Pear

Supplementary Table S9: Formulation 2: L3

Parameter	Lower Limit	Mean value	Upper Limit	CV%	Distribution
blood plasma	0.678	0.693	0.708	0.73	Log-Normal
fraction unbound	0.658	1.00	1.52	15	Log-Normal
$K_{VH/w}$	3846.1	18395	8.80×10^{4}	68.5*	Log-Normal
$K_{STCOR/w}$	669.11	3201	1.53×10^{4}	68.5*	Log-Normal
$K_{VE/w}$	60.873	291	1393.2	68.5*	Log-Normal
$K_{DE/w}$	3.4912	16,7	7.99×10^{1}	68.5*	Log-Normal
D_{VH}	5.82×10^{-4}	7.75×10^{-4}	1.00×10^{-3}	10	Log-Normal
D_{STCOR}	1.64×10^{-7}	7.83×10^{-7}	3.75×10^{-6}	68.5*	Log-Normal
D_{VE}	3.17×10^{-9}	4.21×10^{-9}	4.21×10^{-9}	10	Log-Normal
D_{DE}	3.67×10^{-8}	1.76×10^{-7}	8.41×10^{-7}	68.5*	Log-Normal
Application Time *in vivo*	0.00062	0.00099	0.00160	16.8	Log-Normal
Solvent Residual Volume fraction	0.2257	0.275	0.335	6.8	Log-Normal
Cl liver	4.90×10^{4}	363720	2.70×10^{6}	95.1	Log-Normal
All other parameters				GP value	Log-Normal

Supplementary Table S10: Calculation of the uncertainty (expressed as a %CV) for the parameters used in the models. GP = GastroPlus Software.

**Parameters**	**CV% calculation method**	**Value**	**Distribution**
**Blood: plasma ratio (Rbp)**	Experimental CV or CV from analytical validation if BLQ*	15%	Log-Normal
**Fraction unbound from plasma proteins (Fup)**	Experimental CV or CV from analytical validation if BLQ*	15%	Log-Normal
**Intrinsic clearance in skin (if needed)**	CV T1/2+CV Fu+CV (S9PGL)	CV S9PGL=27%	Log-Normal
**Vehicle/Water partition coefficient ($K_{VE/W}$)**	Experimental CV on ex-vivo concentrations of the distribution study. Choice of the most important CV among all skin layers		Log-Normal
**Stratum corneum/water partition**			Log-Normal

**coefficient ($K_{STCOR/W}$)**			
**Epidermis/water partition coefficient ($K_{VE/W}$)**			Log-Normal
**Dermis/water partition coefficient ($K_{DE/W}$)**			Log-Normal
**Stratum corneum diffusivity (D_{STCOR})**			Log-Normal
**Epidermis diffusivity (D_{VE})**			Log-Normal
**Dermis diffusivity (D_{DE})**			Log-Normal
**Vehicle/Water diffusivity (D_{VH})**	CV GP***	CV GP=10%	Log-Normal
**Application Time (full PBK model)**	CV (expe evapo)+CV GP	CV GP=10%	Log-Normal
**Solvent residual volume fraction**	Experimental CV on vehicle evaporation measurements		Log-Normal
**K evaporation rate**	CV GP	CV GP=10%	Log-Normal
**Reprog organe volume**	CV GP	CV GP=10	PEAR
**Intrinsic clearance in liver**	CV Fu+CV HPGL +CV vol foie (GP,PEAR)+CV expe Cl hepato	CV vol foie=16.8<br>CV HPGL=42+15%	Log-Normal
**Ksys**	CV GP	CV GP=10	Log-Normal
**Kidney volume**	CV GP	19,4	PEAR

**Liver volume**	CV GP	16,8	PEAR
**Intrinsic clearance in Kidney**	CV GP	40	Log-Normal
**Skin volume**	CV GP	10	PEAR
**Adipose volume**	CV GP	10	PEAR
**Venous return volume**	CV GP	10	PEAR
**arterial supply volume**	CV GP	10	PEAR
**All other parameters**	CV GP		Log-Normal

2 Supplementary Figures

Supplementary Figure S1. A) Plasma concentrations in clinical trials after (A) a single dose (Matta et al., 2020) and (B) multiple doses (Matta et al., 2019). (B) Distribution of oxybenzone in different skin compartments 24 h after application of Formulation 1 (F-1), Formulation 2 (F-2), Formulation 3 (F-3), and Formulation 4 (F-4) containing oxybenzone to fresh human skin. The red symbol in (A) denotes a single outliers in the measured values. Values in (C) are the mean ± SD of the amount in µg/cm^2.

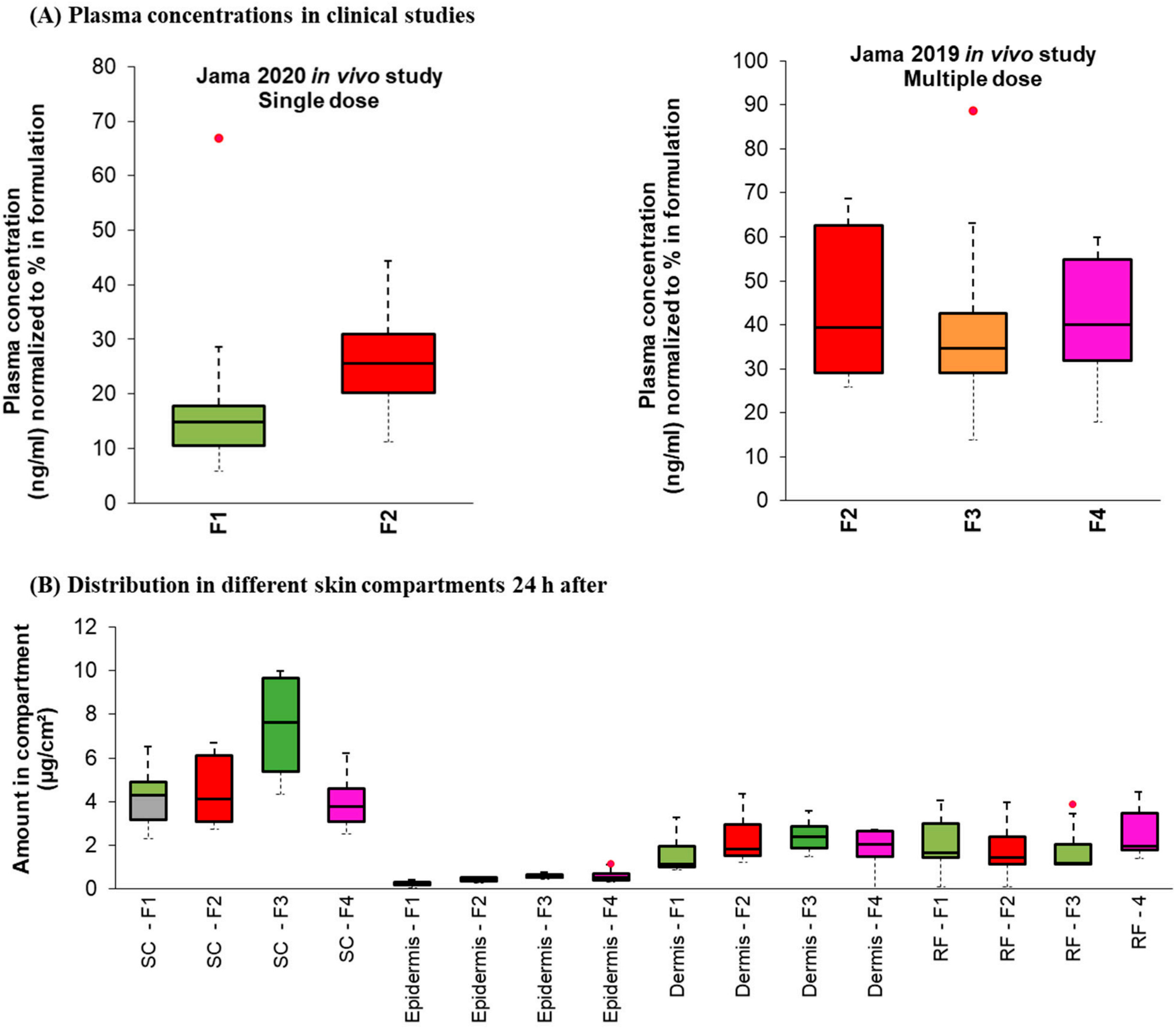

Supplementary Figure S2. Formation of metabolites in primary human hepatocytes incubated with 100 µM oxybenzone. Values are the mean peak area ± SD.

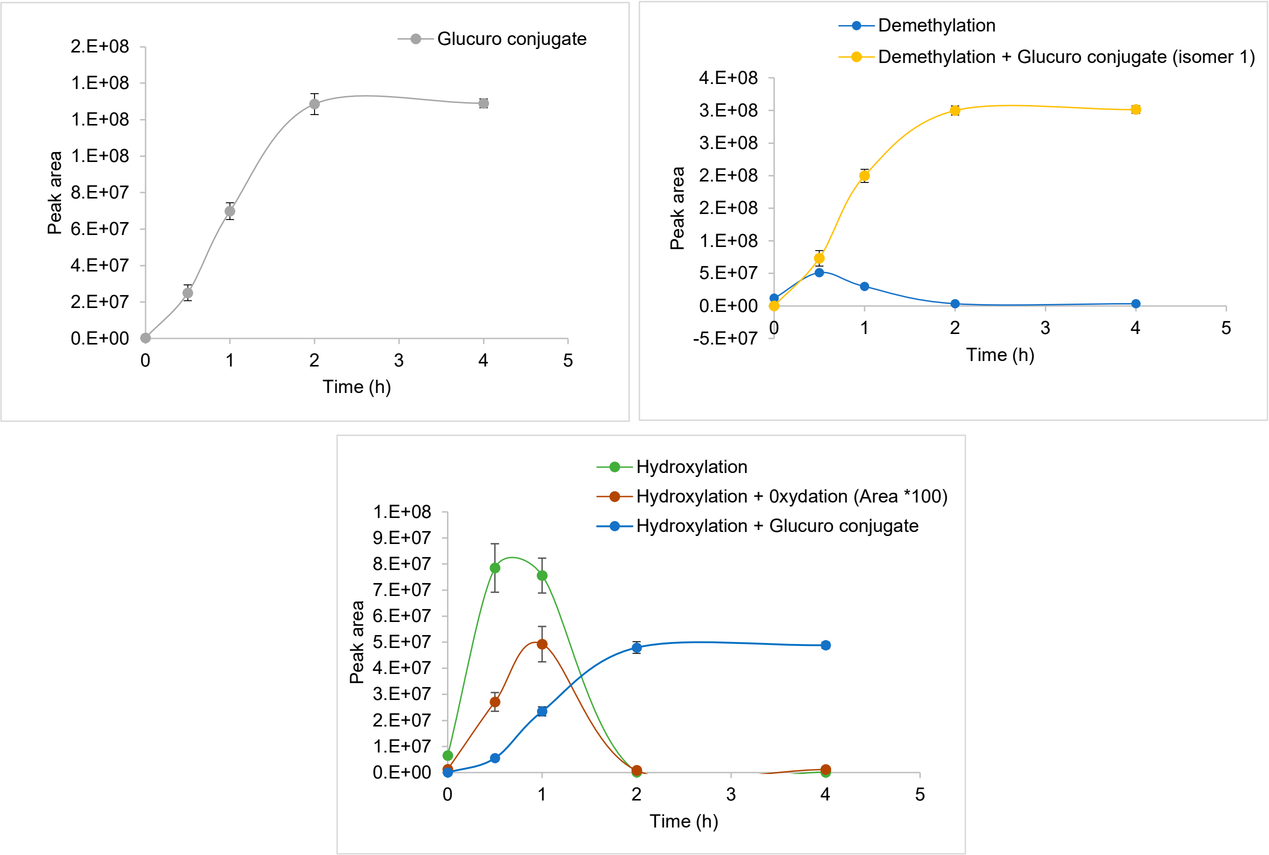

Supplementary Figure S3. Oxybenzone metabolite identification.

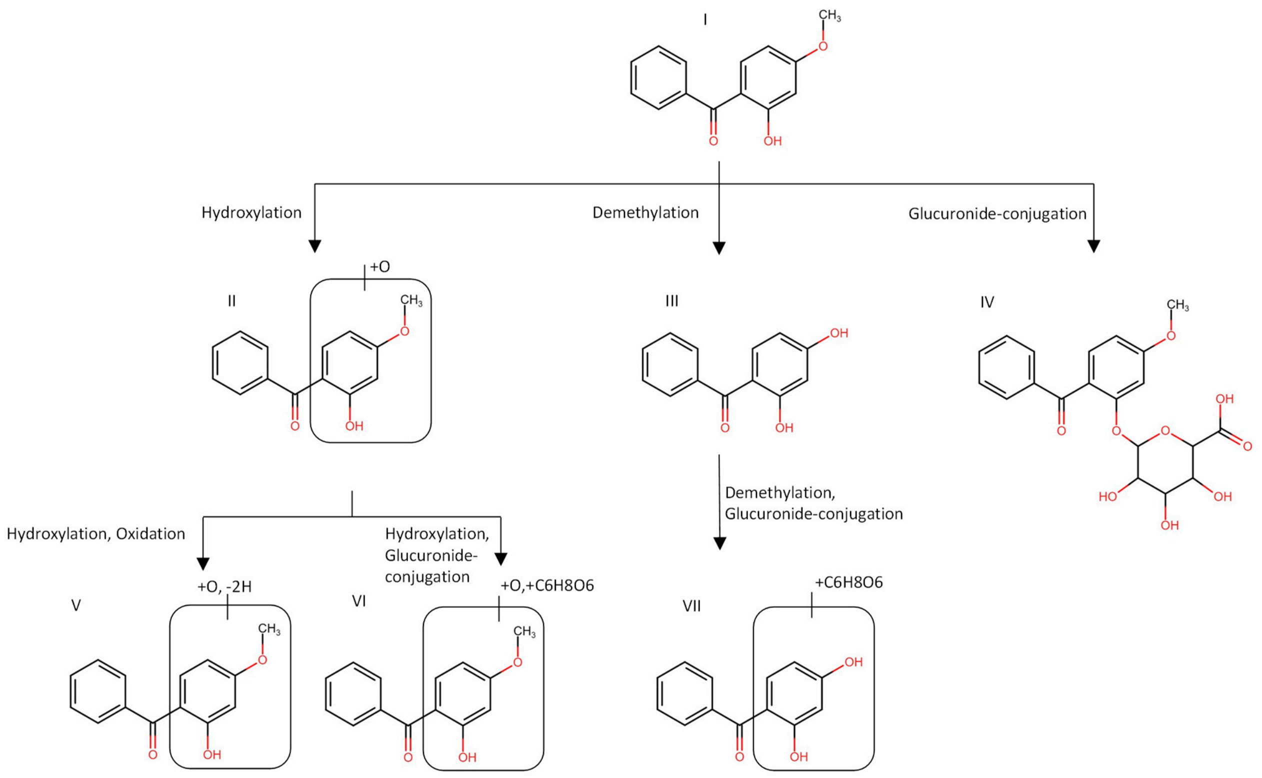

Supplementary Figure 4. Multiple dose population analysis (5o subjects) simulations of plasma concentrations versus time, with the L2 models, from left to right formulations F1 and F2.

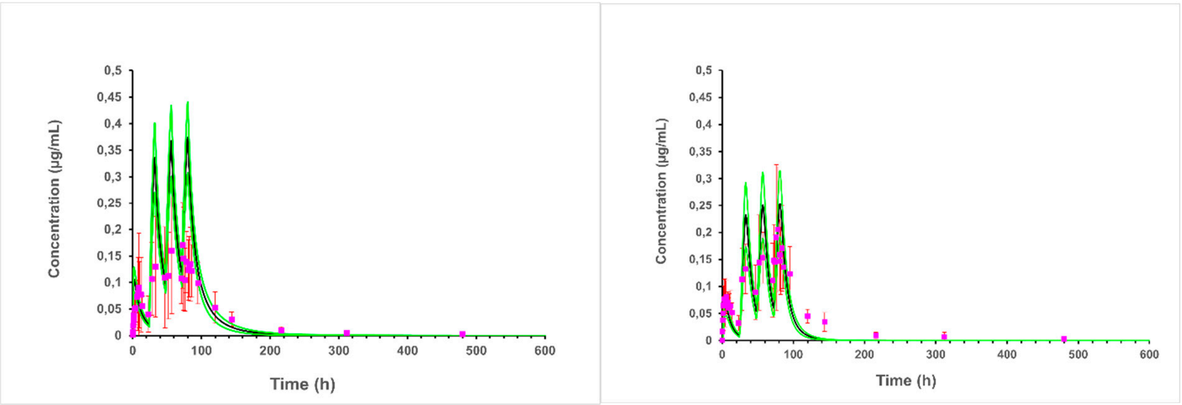

References

Matta, M. K., Florian, J., Zusterzeel, R., Pilli, N. R., Patel, V., Volpe, D. A., Yang, Y., Oh, L., Bashaw, E., Zineh, I., Sanabria, C., Kemp, S., Godfrey, A., Adah, S., Coelho, S., Wang, J., Furlong, L.-A., Ganley, C., Michele, T., & Strauss, D. G. (2020). Effect of Sunscreen Application on Plasma Concentration of Sunscreen Active Ingredients: A Randomized Clinical Trial. *JAMA*, 323: 256-267.

Matta, M. K., Zusterzeel, R., Pilli, N. R., Patel, V., Volpe, D. A., Florian, J., Oh, L., Bashaw, E., Zineh, I., Sanabria, C., Kemp, S., Godfrey, A., Adah, S., Coelho, S., Wang, J., Furlong, L.-A., Ganley, C., Michele, T., & Strauss, D. G. (2019). Effect of Sunscreen Application Under Maximal Use Conditions on Plasma Concentration of Sunscreen Active Ingredients: A Randomized Clinical TrialEffect of Sunscreen Application on Plasma Concentration of Active IngredientsEffect of Sunscreen Application on Plasma Concentration of Active Ingredients. *JAMA*, 321: 2082-2091.

Yu, S., Li, S., Yang, H., Lee, F., Wu, J. T., & Qian, M. G. (2005). A novel liquid chromatography/tandem mass spectrometry based depletion method for measuring red blood cell partitioning of pharmaceutical compounds in drug discovery. *Rapid Commun. Mass Spectrom.*, 19: 250-254.
